# Naphthalene: MAK Value Documentation, addendum – Translation of the German version from 2022

**DOI:** 10.34865/mb9120e10_2ad

**Published:** 2025-06-30

**Authors:** Andrea Hartwig, MAK Commission

**Affiliations:** 1 Institute of Applied Biosciences. Department of Food Chemistry and Toxicology. Karlsruhe Institute of Technology (KIT) Adenauerring 20a, Building 50.41 76131 Karlsruhe Germany; 2 Permanent Senate Commission for the Investigation of Health Hazards of Chemical Compounds in the Work Area. Deutsche Forschungsgemeinschaft, Kennedyallee 40, 53175 Bonn, Germany. Further information: Permanent Senate Commission for the Investigation of Health Hazards of Chemical Compounds in the Work Area | DFG

**Keywords:** Naphthalin, Nase, Lunge, Kanzerogenität, Entzündung, Genotoxizität, Keimzellmutagenität, Hautresorption, naphthalene, nose, lung, carcinogenicity, inflammation, genotoxicity, germ cell mutagenicity, skin absorption

## Abstract

The German Senate Commission for the Investigation of Health Hazards of Chemical Compounds in the Work Area (MAK Commission) has summarized and re-evaluated the data for naphthalene [91-20-3] considering all toxicological end points. Relevant studies were identified from a literature search. The critical effects are the carcinogenic effects in rodents. No data in humans are available for these effects. In carcinogenicity studies, inhalation of naphthalene was found to cause neuroblastomas and adenomas in the noses of rats as well as bronchioloalveolar adenomas and carcinomas in mice. Naphthalene is not mutagenic in vitro, but robust data in vivo are not available. In general, DNA damage is induced in vitro and in vivo mostly at toxic doses. Mechanistic studies suggest that the carcinogenic potential arises from a non-genotoxic mechanism of action. This mechanism is initiated only at high concentrations by severe local inflammation leading to increased cell proliferation. However, a number of uncertainties remain. Thus, at present, a primary genotoxic mechanism of action cannot be ruled out completely for naphthalene. There are no studies from which an effect threshold in humans can be derived. Naphthalene thus remains classified in Carcinogen Category 2. As data for the accessibility of the germ cells are not available, naphthalene remains classified in Germ Cell Mutagen Category 3 B. No sensitizing potential can be derived from the available data. Naphthalene can be absorbed via the skin in toxicologically relevant amounts and remains designated with an “H”.

**Table:** 

**MAK value**	**–**
**Peak limitation**	**–**
**Absorption through the skin (2001)**	**H**
**Sensitization**	**–**
**Carcinogenicity (2001)**	**Category 2**
**Prenatal toxicity**	**–**
**Germ cell mutagenicity (2001)**	**Category 3 B**
	
**BAR (2015)**	**35 µg/l in urine (sum of 1-naphthol and 2-naphthol after hydrolysis)**
**EKA (2021)**	**see Klotz et al. (2022)**
	
CAS number	91-20-3
**1 ml/m^3^ (ppm) ≙ 5.318 mg/m^3^**	**1 mg/m^3^ ≙ 0.188 ml/m^3^ (ppm)**

Note: The substance can occur simultaneously as vapour and aerosol.

Documentation for naphthalene was published in 1995 (Greim 1998) followed by an addendum in 2001 in which the substance was classified in Carcinogen Category 2 and Germ Cell Mutagen Category 3 B (Hartwig 2012).

This addendum re-evaluates the classification of the substance in Carcinogen Category 2 on the basis of recent studies of its genotoxicity and mechanism of action as well as in the light of a new workplace study. 

For all end points, the new studies relevant for the assessment are described.

## 1 Toxic Effects and Mode of Action

The general mode of action of naphthalene was presented in detail in the documentation from 1995 (Greim 1998).

## 2 Mechanism of Action

### 2.1 Genotoxicity

Naphthalene concurrently induces lipid peroxidation and DNA strand breaks in certain cell types in vitro as well as in several organs (Bagchi et al. 1998 a, b, 2000). This finding suggests that the substance causes genotoxic effects via indirect mechanisms such as the formation of reactive oxygen species (ROS).

It was concluded in the addendum from 2001 that naphthalene causes genotoxic effects in vivo (Hartwig 2012). This conclusion can now be elaborated further using the data for genotoxicity available today (see Section 5.6):

- It is postulated that the observed genotoxic effects are caused by cytotoxicity (Brusick 2008; Schreiner 2003; see also Section 5.6).
- In addition to the cytotoxic effects caused by the binding of the reactive metabolites of naphthalene to proteins, another possibility currently under consideration is the formation of ROS by naphthoquinone metabolites via enzyme-catalysed redox cycling reactions. The ROS cause oxidative DNA damage, thereby contributing to the induction of secondary, indirect genotoxic effects of naphthalene (AGS 2018; Bagchi et al. 1998 a; Vuchetich et al. 1996).
- Signs of the progression of carcinogenesis via oxidative stress, inflammation and proliferation were observed in rats after exposure to naphthalene by inhalation (Clewell et al. 2014; see Section 2.2) and in mice after intraperitoneal injection of the substance (Hong et al. 2014).

It was postulated that a direct interaction of naphthalene with DNA should have yielded positive results in the mutagenicity tests in Salmonella typhimurium (Brusick 2008). However, the strains that were tested are used to detect mutagenic effects at guanine or cytosine bases. Therefore, this test would not be able to detect a mutagenic process that acts via changes involving adenine or thymine. In addition, strains susceptible to oxidative damage, namely Salmonella typhimurium TA102, TA104 or Escherichia coli WP2, were not tested. In an HPRT test and a TK^+/–^ test with metabolically competent human lymphocytes, however, mutagenic effects were not detected after exposure to naphthalene up to severely cytotoxic concentrations.

**Fig.1:**
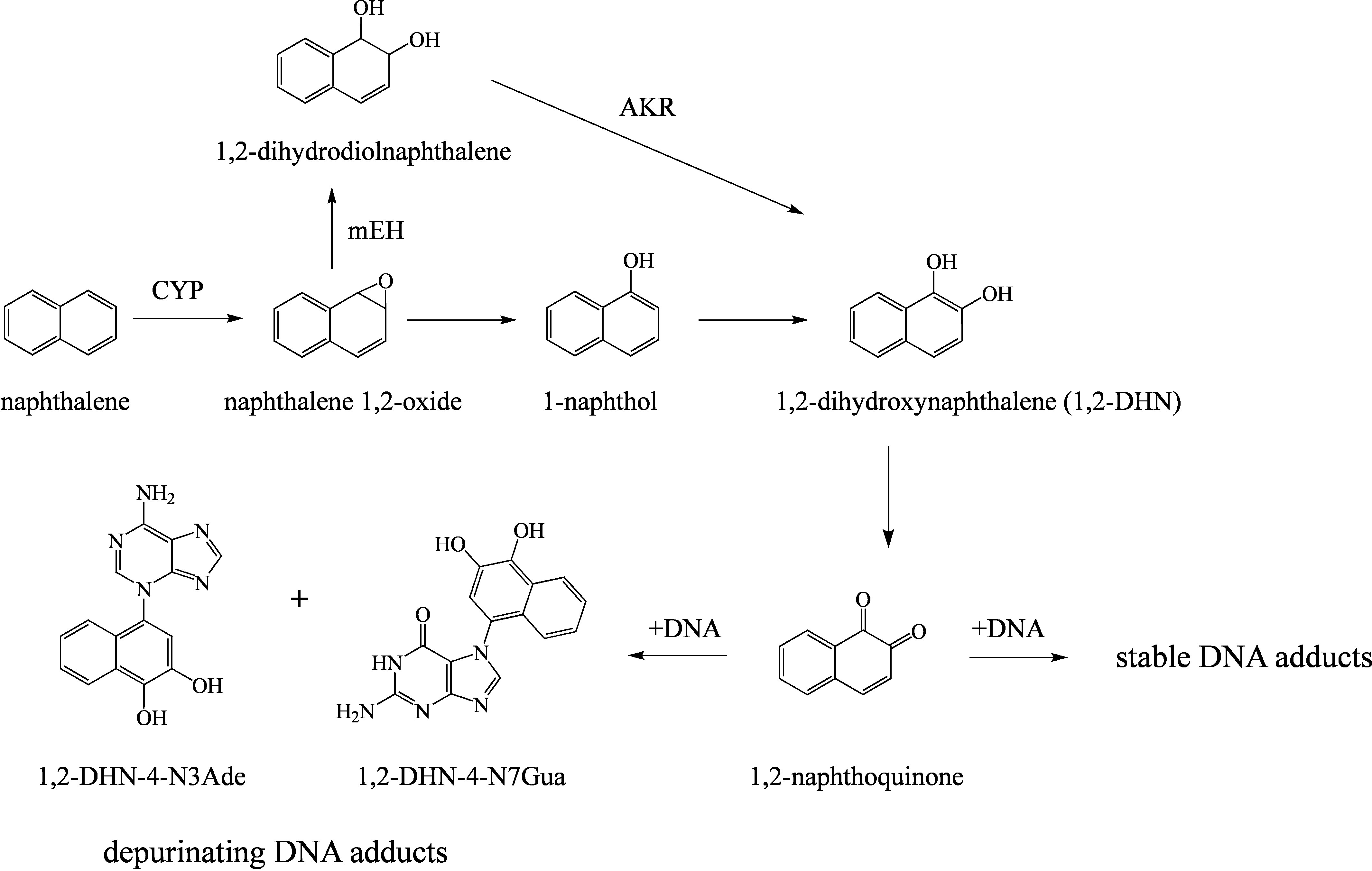
Postulated pathway for the formation of naphthalene–DNA adducts (according to Saeed et al. 2009)

AKR: aldo-keto reductase; CYP: cytochrome P450; mEH: microsomal epoxide hydrolase

Binding of naphthalene to DNA was observed in the lung tissue explants of mice, rats and rhesus monkeys and in the respiratory epithelium, but not in the olfactory epithelium, of the nose of rats (Buchholz 2016; Buchholz et al. 2019; Carratt et al. 2019 a). The possible involvement of a direct genotoxic mechanism in the carcinogenic effects is therefore discussed below:

A study in Sencar mice reported the binding of 1,2-naphthoquinone, a metabolite of naphthalene, to the DNA and the formation of unstable purine base adducts in the skin (Saeed et al. 2009). However, these findings are not regarded as evidence that these types of reactions occur also in the target cells of carcinogenicity and cause mutations there (Bailey et al. 2016). Sencar mice are highly susceptible to skin lesions; for this reason, the Commission does not consider the positive findings obtained in the skin of these mice in tumour promotion studies relevant for the classification (Laube et al. 2019). N7-Guanine adducts are unstable and rapidly undergo depurination to form apurinic (AP) sites. However, these adducts and the AP sites they form seem to have limited mutagenic or carcinogenic potency (Boysen et al. 2009). The postulated metabolic pathway for the formation of adducts is shown in Figure 1 (Saeed et al. 2009).

The mouse skin study did not include an investigation of cytotoxicity (Saeed et al. 2009). Therefore, the result is no evidence of DNA adduct formation in the subtoxic range. Detoxification, for example via glutathione (GSH), can prevent the binding of reactive metabolites to the DNA below cytotoxic concentrations that lead to the depletion of GSH (Bailey et al. 2016). Accordingly, it was shown that the tissue damage was preceded by GSH depletion in the lungs and nose of mice. The lungs, nose and liver of at least 4 male NIH Swiss mice per concentration group were examined after inhalation exposure to naphthalene concentrations of 0, 1.5 or 15 ml/m^3^ for 2 or 4 hours. In the group exposed to 15 ml/m^3^ for 4 hours, damage to the club cells in the distal and proximal airways was observed 4 to 8 hours after exposure and damage to the olfactory nasal epithelium was noticeable after 24 hours. In both tissues, the damage was preceded by a decrease in GSH by more than 90% that reached its maximum level after only 2 hours (Phimister et al. 2004).

In 2 publications, binding to the DNA was observed after in vitro exposure of lung tissue explants from mice, rats and rhesus monkeys and nasal tissue from rats (Buchholz 2016; Carratt et al. 2019 a). However, the studies reported contradictory findings, making them of only limited relevance for the evaluation. Buchholz (2016) found that mice exhibited the highest level of DNA binding activity by a considerable margin (animal strain and sex not specified), followed by rhesus monkeys. Carratt et al. (2019 a), by contrast, found that the binding of naphthalene to DNA did not differ with statistical significance between the 2 species and that rhesus monkeys tended to have higher levels of DNA binding activity. As the study of Buchholz (2016) is available only in the form of a report, this discrepancy is not discussed by Carratt et al. (2019 a), even though the data were generated by the same research group. Both studies found that in rats most of the DNA binding occurred in the lungs and somewhat less in the respiratory and olfactory epithelium of the nose. However, the differences determined by Carratt et al. (2019 a) are not statistically significant and Buchholz (2016) did not provide statistical data. The binding activities observed in monkeys and mice were 20 and 30 times as high, respectively. According to the authors, the similar levels of DNA binding activity found in the nose, the target organ of carcinogenicity, and in the lungs of rats (non-target organ) suggest that the detected adducts are unstable and are rapidly repaired in vivo. This supports the assumption that a non-mutagenic mechanism underlies the formation of nasal tumours in rats (Buchholz 2016; Carratt et al. 2019 a). In general, the authors conclude that the type of DNA binding has yet to be conclusively defined and it may be efficiently eliminated/repaired in vivo (Carratt et al. 2019 a).

Even though the authors use the term “DNA adducts” to describe the DNA binding, only in a single study with explants from rhesus monkeys was the DNA enzymatically digested to deoxynucleosides, thereby providing evidence of covalent DNA binding (Carratt et al. 2019 a). The authors have not yet achieved their objective of identifying the specific DNA adducts (Buchholz 2016). The occurrence of DNA adducts would be evidence of a genotoxic mechanism for tumour formation. However, neither study includes exact data for cytotoxicity. Therefore, these results cannot be used as conclusive evidence that specific adducts form or as mechanistic evidence that the DNA damage is induced only after depletion of the GSH pool and the onset of cytotoxicity.

In another study, pretreatment with naphthalene for 7 days by intraperitoneal injection did not decrease the DNA binding activity (Buchholz et al. 2019).

Negative results obtained in the *Tp53* mutation test in the rat nose, the target tissue of carcinogenicity, support the hypothesis that naphthalene does not induce carcinogenicity via a mutagenic mechanism of action (Bailey et al. 2016). In this study, 10 female and 10 male F344 rats per concentration group were exposed by inhalation to naphthalene concentrations of 0, 0.1, 1.0, 10 or 30 ml/m^3^ for 6 hours a day, on 5 days a week, for 13 weeks and then analysed for CGT to CAT point mutations in *Tp53* codon 271 (equivalent to human *TP53* hotspot mutations in codon 273). No statistically significant effects were found in female rats, while the decrease in mutation frequency observed in male rats at 30 ml/m^3^ was statistically significant in comparison with the values determined in the controls. The authors attributed this finding to increasing cytotoxicity (Meng et al. 2011). However, the analysed transition is not a mutation that would typically be expected to occur via a mechanism involving DNA adduct formation, as established for other polycyclic aromatic hydrocarbons (PAHs) such as benzo[a]pyrene (BaP), or via lesions induced by ROS. Therefore, the negative results of this test for *Tp53* mutations cannot be regarded as evidence that naphthalene lacks mutagenic potential. Studies in workers with exposure to naphthalene in mixtures such as jet fuel or bitumen did not find an increase in DNA damage in the blood lymphocytes. None of the available studies investigated genotoxicity in human respiratory tract cells (Krieg et al. 2012; Nies et al. 2022; see Section 5.6). However, micronuclei were induced in vitro in human lymphoblasts also at non-cytotoxic concentrations (Recio et al. 2012; see Section 5.6.1).

**Summary:** At present, it is not possible to rule out a genotoxic mechanism as (one of) the cause(s) of the carcinogen­icity induced by naphthalene. Numerous findings suggest that the genotoxic effects play a role only at high concentrations and after the onset of cytotoxicity. However, isolated genotoxic effects were observed also in the non-cytotoxic range. It cannot be concluded with certainty whether the DNA binding activity and DNA adducts found in explant cultures of the target tissues of carcinogenicity contribute to the development of carcinogenicity because reliable mutagenicity tests in these tissues are not available. Therefore, at present it is not possible to rule out a primary genotoxic mechanism of action and no threshold can be derived based on the available data.

### 2.2 Gene expression analyses

Ten male and 10 female F344 rats per sex and group were exposed by inhalation to naphthalene concentrations of 0, 0.1, 1, 10 or 30 ml/m^3^ for 6 hours a day, on 5 days a week, for 90 days (Dodd et al. 2012). In another study, RNA was isolated from the respiratory and olfactory epithelium of the nose and the gene expression was analysed. At the low concentration, only few changes in gene expression were observed. There was evidence of oxidative stress in the respiratory epithelium at concentrations of 1 ml/m^3^ and above. At concentrations of 10 ml/m^3^ and above, marked changes in gene expression were detected in the respiratory and olfactory epithelium. These are signs of oxidative stress, inflammation and proliferation. According to the authors, the results are in agreement with both the postulated mechanism of action (cytotoxicity leading to carcinogenicity) and the effect concentrations of the carcinogenicity study published by the NTP (2000). The authors calculated a BMD and BMDL of 6 and 3.7 ml/m^3^, respectively, for the most sensitive changes in gene expression in the olfactory epithelium of female rats and values of 0.4 and 0.3 ml/m^3^, respectively, for those found in the respiratory epithelium of male rats. With a physiologically based pharmacokinetic model (PBPK) for naphthalene using the BMDL for male rats and a safety factor of 3, the authors calculated a NAEC (no adverse effect concentration) of 0.3 ml/m^3^ for continuous environmental exposure of humans (Clewell et al. 2014; Dodd et al. 2012).

Another gene expression study that included an ingenuity pathway analysis investigated male and female C57BL/6 mice that were exposed by inhalation to a naphthalene concentration of 10 ml/m^3^ for 4 hours. The study found that genes that are associated with lung tumour formation were activated in animals of both sexes. “DNA damage” was slightly increased in the females and slightly decreased in the males. “DNA repair” was markedly increased in the males and slightly reduced in the females. The genes included *Cyp2f2 *(cytochrome P450 2f2), *Scg1a1* (club cell secretory protein) in addition to numerous genes that are involved in GSH synthesis and metabolism (*Gpx6*, *Gsta3*, *Mgst1*, *Eef1b2*, *Gsta4*, *Mgst2*, *Gstk1*, *Hnmt*, *Gstm4*). In the same study, cytotoxic changes were observed in the epithelium of the respiratory tract. These were manifest as swelling and vacuolization in the cytoplasm of club cells; a concentration-dependent increase was observed at 5 ml/m^3^ and above. Other cell types in the respiratory tract and the alveolar epithelium remained unchanged. In female mice, swelling of the club cells was more pronounced in the proximal respiratory tract than in the distal respiratory tract (Carratt et al. 2019 b).

### 2.3 Carcinogenicity

Long-term inhalation studies found bronchioloalveolar adenomas and a single carcinoma in the lungs of B6C3F1 mice and adenomas in the respiratory epithelium of the nose and neuroblastomas in the olfactory epithelium in F344 rats (see Section 5.7).

The mechanisms of action discussed below suggest that there may be a threshold for carcinogenicity. However, conclusive evidence is not available.

After inhalation, the metabolism of naphthalene begins in the respiratory tract, forming the reactive metabolites 1,2-dihydroxynaphthalene, 1,4-dihydroxynaphthalene, 1,2-naphthoquinone, 1,4-naphthoquinone and possibly also 1,2-di­hydroxy-3,4-epoxy-1,2,3,4-tetrahydronaphthalene. The exact site (upper or lower respiratory tract) and rate of these reactions vary according to species and tissue. The metabolites are deactivated by conjugation and eliminated as mercapturic acid, glucuronic acid or as sulfate. Higher naphthalene concentrations lead to the depletion of intracellular GSH and glucuronic acid, with the result that the reactive intermediates are no longer detoxified and bind to cellular macromolecules (AGS 2018; Buchholz 2016; Buchholz et al. 2019; Carratt et al. 2019 a; Greim 1998).

A gene expression study in the nasal epithelium of rats after inhalation exposure to naphthalene confirmed the postulated mechanism of action that cytotoxicity leads to carcinogenicity. The results indicate oxidative stress, inflammation and proliferation at concentrations which concur with the effect concentration in the carcinogenicity study of the NTP (2000) (Clewell et al. 2014; Dodd et al. 2012; see Section 2.2).

A Naphthalene State-of-the-Science Symposium was held to investigate the carcinogenic mechanism of action of naphthalene in rats and mice (Belzer et al. 2008; Bogen et al. 2008; Brusick et al. 2008; North et al. 2008). The panel of experts drew the following conclusions:

- The formation of tumours in the nose of rats is greatly enhanced, if not enabled, by histologically related focal cellular proliferation.
- The incidence of lung tumours in mice was increased at a concentration cytotoxic to the lungs of 30 ml/m^3^, but not at the less markedly cytotoxic concentration of 10 ml/m^3^. This suggests a NOAEC (no observed adverse effect concentration) for tumour development in mice of 10 ml/m^3^.
- Cytotoxicity induced by naphthalene requires metabolic activation.
- The metabolic activation of naphthalene varies markedly according to tissue type and species.
- The anatomy and physiology of the target tissues of rodents, non-human primates and humans are sufficiently well understood to parameterize species-specific PBPK models for effects in the nose and lungs.

The panel of experts concluded that the following information was lacking for the estimation of a cancer risk in humans (Belzer et al. 2008; Bogen et al. 2008; Brusick et al. 2008; North et al. 2008):

- the cytotoxic metabolites of naphthalene, their mechanism of action and data for effects induced in the tissue of monkeys and humans in the low-dose range
- mechanistic and toxicokinetic inhalation studies in mice, rats and monkeys to better understand the uptake and metabolism of naphthalene in the upper respiratory tract
- in vivo studies to validate the PBPK models for monkeys exposed to naphthalene by inhalation in conjunction with cytotoxicity studies
- in vivo studies to validate a human PBPK model

Another consideration is that cell proliferation and inflammation in rodents do not coincide with tumour formation in the organs. Therefore, cell proliferation was more marked in the nose (non-target organ) of mice than in the lungs (target organ) (NTP 1992; see Section 5.7).

#### 2.3.1 Human relevance of the nasal tumours in the rat

Inhalation exposure to naphthalene caused malignant neuroblastomas in the olfactory epithelium of female rats and adenomas in the respiratory epithelium of male rats (NTP 2000, see Section 5.7).

Naphthalene induced cytotoxic effects in the nose of rats and mice, but was carcinogenic only in the nose of rats. A study investigating absorption rates in the upper respiratory tract in both species found higher levels of absorption in mice than in rats. The authors concluded that absorption rates are not associated with carcinogenicity (Morris 2013; Morris and Buckpitt 2009).

The CYP2F enzyme catalyses the oxidation of naphthalene in the nose of rats, while CYP2A13 is found in the human respiratory tract. As a result, there are specific 1,2-naphthoquinone metabolites that form in rodents but do not occur in humans. In rats, exposure to high concentrations of naphthalene leads to the depletion of GSH, making the metabolite 1,2-naphthoquinone available for other conjugations. In the rat nose, it is proposed that a naphthoquinone imine is formed from 1,2-naphthoquinone via a species-specific and tissue-specific aryl amidase. However, there is no evidence to support this suggestion. The carcinogenic effects of alachlor and phenacetin are likewise attributed to a quinone imine. These substances cause adenomas and carcinomas in the nose of rats, but do not induce similar carcinogenic effects in humans. On the basis of this mechanism of action and the limited epidemiological data for carcinogenic effects induced by naphthalene in humans, the authors consider that the tumours in the rat nose are not relevant to humans (Piccirillo et al. 2012).

By contrast, statistically significant differences were not found between the naphthalene protein adducts that formed in the nasal epithelium of 8 male Sprague Dawley rats incubated in vitro with 250 µM ^14^C-naphthalene and those detected in 8 male rhesus monkeys. Many of the bound proteins were identified in the subsequent analysis: in rats, for example, these were structural proteins (actin), catalytic enzymes (ATP synthase) and proteins critical for the correct folding and functioning of other proteins such as heat shock proteins in rats and protease inhibitors in rhesus monkeys (DeStefano-Shields et al. 2010). Protein binding was found to be somewhat lower in comparison with the binding activity reported by an earlier in vitro incubation study with the target tissue of mice (the lung tissue) (Cho et al. 1994).

Therefore, the quantitatively similar levels of protein binding observed in rhesus monkeys and rats contradict the marked differences shown by the data for metabolism. An explanation for this discrepancy has yet to be found (Buckpitt et al. 2013).

A different situation presents itself with respect to cytotoxicity and GSH depletion in the nose of rhesus monkeys and rats. Rats seem to be much more sensitive in this case. In a study that is available only in the form of an abstract, exposure of nasal epithelial tissue explants of rhesus monkeys to naphthalene in vitro for 3 hours led to slight GSH depletion only at the highest concentration of 500 µM, but not at 10, 50 or 100 µM (Van Winkle et al. 2014). Based on the predicted concentrations in nasal epithelial tissue of mice, 500 µM is probably equivalent to an inhaled concentration greater than 10 ml/m^3^ (Morris 2013). The validity of this comparison is limited because the conversion of concentrations in vitro to inhalation concentrations contains inaccuracies.

In contrast, GSH was depleted in the respiratory/transitional and olfactory epithelium of rats at statistically significant levels after exposure for 6 hours to naphthalene concentrations of 1 ml/m^3^ and above (Cichocki et al. 2014).

It is likely that numerous enzymes and metabolites are involved in the development of the carcinogenicity caused by naphthalene. On the basis of the study findings presented in this addendum, it cannot be ruled out at present that the carcinogenic effects induced by naphthalene in the nose of rats may be relevant to humans.

#### 2.3.2 Human relevance of the lung tumours in the mouse

A particularly high level of CYP activity was found in the club cells of the lungs of mice; at the same time, the levels of GSH and glucuronic acid are lower in these cells than, for example, in liver cells. As a result, toxic effects may occur in the lungs even after exposure to relatively low naphthalene doses (Greim 1998). After in vitro treatment with naphthalene of club cells from the lungs of mice, 1,2-naphthoquinone was the major metabolite covalently bound to proteins (Zheng et al. 1997). Recent studies confirm that local toxicity in the respiratory tract of mice is mediated by CYP2F enzymes:

A study with knockout mice (C57BL/6 mice) revealed that the loss of CYP2F2 led to resistance against the toxicity induced by naphthalene in the lungs, but not in the olfactory mucosa of the nose. Except for CYP2F2, CYP enzymes are expressed at similar levels in *Cyp2f2*(–/–) mice and wild type mice. *Cyp2f2*(–/–) mice metabolize naphthalene more slowly; this was determined in vitro by analysing the levels of naphthalene-GSH in microsomal extracts of the lungs (~160-fold), nasal olfactory mucosa (~16-fold) and liver (~3-fold) and in vivo based on the levels of naphthalene-GSH and unmetabolized naphthalene in the plasma after intraperitoneal administration of 300 mg/kg body weight (over a period of 0 to 8 hours after injection). In wild type mice, the necrosis and detachment of club cells and ciliated airway epithelial cells were observed in the lungs of all animals 24 hours after intraperitoneal injection of 200 or 300 mg/kg body weight (n = 6/genotype and dose). In 1 of 6 *Cyp2f2*(–/–) mice, slight vacuolization was found in several club cells only at the high dose. At both doses, the olfactory nasal epithelia were damaged to a similar degree in the 2 mouse genotypes; lesions were not detected in the liver (Li et al. 2011).

There are primarily quantitative differences in the metabolism of naphthalene between humans and the different rodent species (see also Section 3.2).

After inhalation exposure to naphthalene concentrations of up to 100 ml/m^3^ for 4 hours, concentration–dependent toxic effects occurred in the respiratory tract of mice, but not of rats, at concentrations of 2 ml/m^3^ and above (Greim 1998).

It is likewise assumed that metabolic activation is higher and the detoxification of the metabolites in the lungs is lower in mice than in humans. These findings are based on studies in humans that found that the CYP2F family of enzymes is less active in humans than in rodents, particularly in mice (Cruzan et al. 2009; Greim 1998; Hartwig 2012).

However, studies in Escherichia coli strains expressing different recombinant human CYPs suggest that human CYP2A13 exhibits a comparatively high level of activity for the hydroxylation of naphthalene to 1-naphthol and 2-naphthol. In humans, CYP2A13 is active primarily in the respiratory tract with a high level of activity in the nasal mucosa followed by the lungs and trachea (Fukami et al. 2008).

CYP2F1 and CYP2A13 humanized mice were used to demonstrate that human enzymes contribute significantly to the toxicity induced by naphthalene in the respiratory tract (Li et al. 2017). In humans, CYP2A13 and CYP2F1 are expressed primarily in the respiratory tract (Carr et al. 2003; Su et al. 2000; Weems and Yost 2010; Zhang and Ding 2008). Additional studies with knockout mice found that hepatic CYP enzymes also play a role in the toxicity induced by naphthalene in the respiratory tract, but to a much lesser extent (Kovalchuk et al. 2017, 2019).

The enzymatic opening and thus deactivation of the epoxide ring of the naphthalene 1,2-epoxide that initially forms is probably more efficient in humans than in mice. This is supported by an in vitro study that found that the metabolism of naphthalene via human liver microsomes led to a ratio of naphthalene *trans*-1,2-dihydrodiol to 1-naphthol of 8.6 compared with a ratio of 0.4 if the liver microsomes from phenobarbital-treated mice were used (IARC 2002).

Overall, the data for metabolism in mice and humans suggest that humans are less susceptible to the toxic effects of naphthalene in the lungs. However, there is a lack of more specific information on the affected cell types in the respiratory tract, in which the enzyme activities may clearly differ between the species.

#### 2.3.3 Summary

In spite of species differences in the metabolic activation of naphthalene, the nasal and lung tumours found in rodents may be relevant to humans. It is postulated that the secondary genotoxic effects induced at high concentrations mani­fest themselves as carcinogenic effects only with a concurrent increase in cell proliferation and inflammation. As a result, carcinogenic effects can be avoided by preventing inflammation. However, some of the studies that investigated genotoxicity (see Section 5.6 and 2.1) found evidence of a primary genotoxic mechanism. Therefore, it is not clear whether it is possible to derive a threshold for protection against carcinogenicity.

### 2.4 Other investigations

The role of CYP-mediated metabolism and the electrophile-sensitive transient receptor potential ankyrin 1 receptor (TRPA1) in mediating the sensory irritation response to inhalation exposure to naphthalene was investigated in female C57BL/6J mice during exposure for 15 minutes to a naphthalene concentration of 7 ml/m^3^ via plethysmographic determination. In comparison with the values found in untreated wild type mice, the sensory irritation response was reduced to less than a third in animals pretreated with the CYP inhibitor metyrapone and completely absent in *Trpa1* knockout mice. This supports the hypothesis that the sensory irritation response to naphthalene is mediated by the TRPA1 receptor via its metabolites (Lanosa et al. 2010).

Regardless of whether the route of administration was inhalation or intraperitoneal injection, acute exposure of rats (n = 3) to naphthalene caused damage to the olfactory epithelial tissue of the nose. After intraperitoneal injection of naphthalene doses of 0, 25, 50, 100 or 200 mg/kg body weight in corn oil, the damage was distributed evenly throughout the olfactory epithelium. After inhalation exposure to naphthalene concentrations of 0, 3.4 or 23.8 ml/m^3^ for 4 hours, the damage occurred mainly in the medial meatus. Due to higher levels of CYP enzyme activity, naphthalene was metabolized more rapidly after incubation in vitro with microsomes from the olfactory epithelium than after incubation with those from the respiratory epithelium. Therefore, the amount of damage that develops in the nose probably correlates with the amount of air that reaches the different regions of the nose and the metabolic competence of the respective tissues. The main metabolites were GSH conjugates of naphthalene-1,2-epoxides and primarily 1R-hydroxy-2R-glutathionyl-1,2-dihydronaphthalene, which is formed from 1R,2S-naphthalene epoxide (Lee et al. 2005).

A single intraperitoneal injection of a naphthalene dose of 200 mg/kg body weight in corn oil in male C57BL/6 mice (no exact data for the number of animals) increased the number of cells in the bronchoalveolar lavage fluid (BALF) and caused epithelial denudation and pulmonary hyperreactivity (methacholine test) in the lungs after 12, 24, 48 or 72 hours (Karagiannis et al. 2012).

Groups of 5 to 6 mice were given a single intraperitoneal injection of naphthalene in olive oil at dose levels of 0, 50, 75, 100 or 200 mg/kg body weight. After 24 hours, changes were observed in the metabolome of the BALF and the lungs with respect to lipid peroxidation, membrane damage and disruptions in energy supply, particularly at the 2 high doses (Hong et al. 2014).

Two follow-up studies analysed the metabolome of the lungs, liver, kidneys, blood and BALF of male ICR mice after being given a naphthalene dose of 300 mg/kg body weight by intraperitoneal injection either as a single injection or following administration of a naphthalene dose of 200 mg/kg body weight and day in olive oil for 7 days. Histopathological damage was only observed in the lung tissue of the animals that received a single dose. In the lungs, changes were observed in the metabolites of energy metabolism. After acute exposure, the damage to the tissues was accompanied by increased acetone levels in the BALF and a changed spectrum of metabolites such as cholesterol, phosphorylcholine-containing lipids (lyso-phosphatidylcholines and sphingomyelins) and fatty acids in the lung tissue. The authors suggest that the tolerance to lung tissue damage may be explained by improved membrane flexibility and antioxidative mechanisms resulting from the induction of GSH and succinate that was observed only after repeated administration and changes in diacyl-phosphatidylcholine and plasmenylcholine. The finding that the metabolome of the kidneys and liver was only slightly affected by exposure to naphthalene is consistent with the lack of tissue damage (Lee et al. 2018; Lin et al. 2015).

Female and male mice were used to study the contribution of the metabolites formed by microsomal epoxide hydrolase (mEH) to the induction of cytotoxicity in the lungs. Mice that were either deficient (mEH-KO) or wild type (mEH-WT) for mEH were exposed by inhalation to naphthalene concentrations of 5, 10 or 20 ml/m^3^ for 4 hours. All mice had swollen and vacuolized cells in the intra- and extrapulmonary airways at concentrations of 5 ml/m^3^ and above. Although mEH-KO mice had less damage in the extrapulmonary airways than mEH-WT mice, damage did occur. In the intrapulmonary airways, the site of tumour formation after inhalation exposure of mice (NTP 1992), mEH-KO mice were more susceptible to naphthalene-induced cytotoxicity than mEH-WT mice at 20 ml/m^3^. The same finding was obtained in male mice also at a concentration of 5 ml/m^3^. The authors concluded that mEH-generated metabolites were not solely responsible for the toxicity and carcinogenicity induced by naphthalene in the respiratory tract of mice. When the differences were analysed by sex, more GSH-conjugated metabolites (mEH-independent) were generated in the intrapulmonary airways in male (mEH-WT and mEH-KO) than in female mice. Female mice were slightly more susceptible to naphthalene-induced cytotoxicity, both in the intra- and extrapulmonary airways (Carratt et al. 2016).

In a mouse model for fibrosis and obstructive lung disease, 14 weekly intraperitoneal injections of naphthalene induced damage to the club cells and regional hyperproliferation of epithelial progenitor cells in the lungs, leading to fibroblast proliferation and peribronchial collagen deposition (fibrosis). This is associated with the induction of fibrogenic cytokines TGFβ (transforming growth factor beta) and CTGF (connective tissue growth factor) and an increase in the total collagen content in the lungs (Aoshiba et al. 2014). However, there was no evidence that naphthalene induces epithelial-mesenchymal transitions that may facilitate the development of obstructive lung disease (Watanabe et al. 2016).

Naphthalene was found to have biological activity in 20 of 894 in vitro tests included in the ToxCast database of the US EPA. All 20 of the tests that yielded positive results were carried out with human liver cell lines (Bailey and Rhomberg 2020; US EPA 2020). However, 15 of the tests with positive results included notices that the studies may be of limited validity. The remaining 5 tests reported the activation of CYP2B6, CYP3A4, retinoid-X-receptor (2 tests) and oestrogen receptor α. However, also negative results are available from several tests that investigated these cellular proteins. Therefore, a single positive result cannot be considered strong evidence that the substance induces a specific effect in this cellular target. Naphthalene was not detected by NMR-spectroscopic analysis in 1 of 3 samples immediately after dissolution in DMSO (NIH 2022). As a result, it is not clear whether the inactive tests are to be attributed to a lack of activity of naphthalene or a lack of substance in the sample. The data for naphthalene must therefore be considered with caution.

The naphthalene metabolite 1,2-naphthoquinone, but not 1,2-dihydroxynaphthalene, disrupts the catalytic activity of protein kinase C in vitro, which is essential for cellular signal transduction and is involved also in cell proliferation (Yu et al. 2002 b).

1,2-Naphthoquinone and 1,4-naphthoquinone inhibited human topoisomerase II alpha, which is involved in DNA transcription (Gurbani et al. 2012).

### 2.5 Summary of the mechanisms of action

Although recent studies and reviews suggest that the carcinogenic effects of naphthalene are caused by a non-genotoxic mechanism of action, the extent to which the genotoxic effects of naphthalene contribute to the development of carcinogenesis has yet to be conclusively explained. CYP-mediated oxidation of naphthalene yields the 1,2-epoxide and numerous other metabolites, some of which are reactive. They are detoxified and excreted mainly after conjugation with glutathione, glucuronic acid or sulfate. Higher naphthalene concentrations lead to the depletion of intracellular GSH, allowing the reactive metabolites to bind to cellular macromolecules. This covalent binding occurs in tissues with high levels of CYP activity. The club cells in the lungs of mice and the nasal epithelium of rats were found to be particularly sensitive. Covalent binding is observed in the liver and kidneys, but also in the lungs of other species, but the substance is less toxic at these sites than in the lungs of mice.

The mechanism of action proposed for tumour formation via the induction of a strong local inflammatory response by cytotoxic metabolites of naphthalene has not been fully proven. This response occurs only at higher concentrations as a result of the binding of the metabolites to proteins after the depletion of GSH.

However, micronuclei were induced in vitro also at non-cytotoxic concentrations. As DNA binding does occur and no mutagenicity tests in the target tissue of carcinogenicity are available, a genotoxic mechanism of action cannot be ruled out completely and an effect threshold cannot be derived on the basis of the available data.

## 3 Toxicokinetics and Metabolism

### 3.1 Absorption, distribution, elimination

In animal studies, naphthalene was readily absorbed orally, dermally and by inhalation (Greim 1998; Hartwig 2012). The same is assumed for humans based on the few data available (AGS 2018; SCOEL 2010).

Blood:air partition coefficients of 700 to 760 were determined for rats (Morris and Buckpitt 2009). Mice absorb larger amounts of the substance by inhalation than rats (mice 0.5 ml/m^3^: 90%; rats 1 ml/m^3^: 50%). In both species, the percentage absorbed decreased with an increase in the concentration. Uptake was markedly decreased in animals pretreated with 5-phenyl-1-pentyne, an inhibitor of CYP enzymes, which metabolically activate naphthalene (Morris 2013; Morris and Buckpitt 2009). A deposition model for the human respiratory tract after exposure by inhalation found a deposition of 12% to 34% in the extrathoracic airways and 66% to 87% in the tracheobronchial airways at a respiratory minute volume of 15 to 60 l/min (AGS 2018; Zhang and Kleinstreuer 2011).

A study investigating dermal absorption in test persons is available. Groups of 5 female and 5 male test persons were exposed by applying 1 ml of a jet propulsion fuel containing naphthalene (JP-8; 0.3% naphthalene) to both forearms (total area of 20 cm^2^; in air-tight exposure chambers). Absorption of the substance into the skin was determined by tape-strip analysis, and penetration by the course of the naphthalene concentration in the blood. A permeability constant Kp of 5.3 × 10^–5^ cm/h was determined based on the kinetics and the blood volume. A flux of 0.159 μg/cm^2^ per hour was calculated from the exposure concentration (Kim et al. 2006). In addition, in vitro studies were carried out in the pig ear skin model and in human skin with JP-8 fuel that contained naphthalene. Fluxes of 0.376 and 0.451 μg/cm^2^ per hour, respectively, were determined (Kanikkannan et al. 2001 a, b). Other aromatic hydrocarbons found in JP-8 fuel may have had a carrier function.

The 2001 addendum included in vitro permeability constants (cm/h) for the abdominal monkey skin of 5.12 × 10^−3^ in acetone, 6.31 × 10^−3^ in acetone plus artificial sweat and 1.87 × 10^−3^ in acetone plus lubricating oil. The flux (converted from data expressed in nmol/cm^2^/h) was 0.035 ± 0.028 μg/cm^2^ per hour (in lubricating oil) and 0.130 ± 0.051 μg/cm^2^ per hour (in acetone plus artificial sweat) (Hartwig 2012).

The urine is the main excretory pathway for radioactively labelled naphthalene (Greim 1998; Hartwig 2012). In rats, about 77% to 93% is excreted with the urine, 6% to 14% with the exhaled air and about 7% with the faeces. The elimination half-life is 48 to 72 hours (AGS 2018). In humans, the substance is likewise excreted rapidly with the urine (Klotz et al. 2018 a; SCOEL 2010).

### 3.2 Metabolism

The metabolism of naphthalene was presented in detail in the MAK documentation published in 1995 and the BAT documentation from 2016. Naphthalene is metabolized mainly in the liver, but also in the kidneys, the respiratory tract and nasal epithelium (Greim 1998; Klotz et al. 2018 a).

The metabolism of naphthalene involves a multi-step process. The first step involves oxidation to the unstable 1,2-naphthalene epoxide, which can be catalysed by a variety of CYP enzymes. The reactive epoxide can either form covalent bonds directly with cellular macromolecules, be detoxified to mercapturic acids by GSH conjugation or undergo further modification. 1-Naphthol and 2-naphthol may form by spontaneous rearrangement or 1,2-dihydrodiolnaphthalene by enzymatic hydration. These are excreted with the urine after glucuronidation or sulfation. Further oxidation of the naphthols or dihydrodiol catalysed by CYP enzymes or aldo-keto reductase may lead to the formation of 1,2-dihydroxynaphthalene or 1,4-dihydroxynaphthalene (1,2-DHN and 1,4-DHN). These, in turn, may be converted to highly reactive naphthoquinones (Bailey et al. 2016; Greim 1998; Hartwig 2012; Klotz et al. 2018 a). 1,2-Dihydroxy-3,4-epoxy-1,2,3,4-tetrahydronaphthalene (diol epoxide) is likewise formed (ATSDR 2005). The naphthoquinones and the diol epoxide are regarded as toxic metabolites (AGS 2018; Bailey et al. 2016).

Detoxification via GSH, sulfation or glucuronidation are particularly relevant at lower naphthalene concentrations; high naphthalene concentrations may lead to the depletion of GSH, activated sulfate or glucuronic acid. This enables the formation of reactive metabolites (Bailey et al. 2016; Greim 1998).

#### 3.2.1 Species-specific differences in metabolism

The relative amounts of intermediate metabolites, the formation of reactive metabolites and the occurrence of the different metabolic pathways vary according to species and tissue and give rise to specific forms of toxicity (IARC 2002; Preuss et al. 2003). As an example, the 1,2-epoxide that forms in the first step may occur in 2 stereoisomeric forms, 1R,2S-epoxide and 1S,2R-epoxide; however, their ratio differs by species. The 1R,2S-epoxide is found in the lungs of mice as well as the nose of rats, mice and hamsters and correlates with the toxicity occurring there. By contrast, mainly the 1S,2R-epoxide is found in the lungs of rats, hamsters, monkeys and humans (Buckpitt et al. 1992; Cruzan et al. 2009).

##### 3.2.1.1 Rodents

Irrespective of the route of absorption, the club cells in the lungs are the target cells of the toxicity in mice, while the nasal epithelium is the target tissue of the toxicity in rats. Particularly the CYP2F enzymes are regarded as responsible for the formation of toxic metabolites in both species of rodent. Therefore, knockout studies with mice revealed that CYP2F2 is essential for the induction of damaging effects in the lungs by naphthalene, whereas CYP1A1 and CYP1A2 do not play a major role (Genter et al. 2006; Li et al. 2011). Studies in knockout mice revealed that naphthalene is, however, bioactivated also by enzymes other than CYP2ABFGS enzymes in the lungs (Kovalchuk et al. 2017). Other studies in knockout mice found that reduced clearance by hepatic CYP enzymes leads to increased naphthalene toxicity in the respiratory tract (Kovalchuk et al. 2019).

The toxic effects induced by naphthalene in the nose of mice were caused by CYP2A5 (CYP1A1, 1A2 and 2F2 do not seem to play a major role) (Hu et al. 2014; Li et al. 2011). This is contradictory to the findings of a study in which pretreatment with the CYP2F inhibitor 5-phenyl-1-pentyne provided protection against the damage induced by naphthalene in the olfactory mucosa of the nose of mice (Genter et al. 2006).

Repeated administration of naphthalene influenced the expression of enzymes that metabolize naphthalene in rodents. After rats were given naphthalene doses of 200 mg/kg body weight by intraperitoneal injection for 7** **days, the expression of CYP2F and CYP2B and the expression of CYP reductase in the pulmonary epithelium were reduced with statistical significance. However, the covalent binding of metabolites to cellular proteins was not decreased (Lakritz et al. 1996). The expression of GSH *S*-transferases in the liver was increased by 68% in mice given naphthalene doses of 200 mg/kg body weight for 7** **days. This increase was not observed in the lungs (Mitchell et al. 2000).

##### 3.2.1.2 Humans

After exposure of humans to naphthalene, the metabolites 1-naphthol and 2-naphthol, 1,2-DHN and 1-naphthyl mercapturic acid were recovered in the urine; 1,2-DHN constituted the largest fraction (Klotz et al. 2018 b, 2019). This agrees with the finding that 1,2-dihydrodiol, 1-naphthol and 2-naphthol form in vitro in a ratio of 100:10:1 in the presence of human liver microsomes (Cho et al. 2006).

Human CYP2F1 exhibited only low activity towards naphthalene in vitro. However, human CYP2A13, which showed the highest activity in the nose followed by the respiratory tract, is likewise able to metabolize naphthalene (Carr et al. 2003; Fukami et al. 2008; Su et al. 2000; Weems and Yost 2010; Zhang and Ding 2008). The enzymes contributed significantly to the toxic effects induced by naphthalene in the respiratory tract of hemizygous *CYP2F1* and *CYP2A13*-humanized mice. However, the toxic effects were weaker than those found in wild type mice (Li et al. 2017).

In vitro, the amount of naphthalene metabolized in the lung tissue of mice is about 3, 8 and 100 times as high as that metabolized in the lung tissues of hamsters, rats and monkeys (IARC 2002).

The ratio of 1,2-dihydrodiol to 1-naphthol was 8.6 after the metabolism of naphthalene in vitro by human liver microsomes and 0.4 after metabolism by the liver microsomes from mice treated with phenobarbital. Therefore, the deactivation of the epoxide of the initially formed metabolite naphthalene 1,2-epoxide by enzymes is much more efficient in humans than in mice (IARC 2002). In a comparison of rats and humans, mainly 1,2-dihydrodiol formed after incubation of naphthalene in vitro with a pool of human liver microsomes from 150 patients, while mainly 1-naphthol was found after incubation with the liver microsomes of rats (Wang et al. 2020).

##### 3.2.1.3 Rhesus monkeys

An in vitro study published only as an abstract that investigated the metabolism of naphthalene in lung sections from newborn rhesus monkeys found the degree of naphthalene metabolism was highest in the intermediate bronchiolar respiratory tract and lowest in the respiratory bronchioles (no other details). The extent of metabolism in monkeys was lower than that in rodents and took place mainly in the parenchyma. The amount of GSH conjugates and diols formed corresponded with the levels of covalently bound naphthalene metabolites (Hartwig 2012).

### 3.3 Summary

Naphthalene is absorbed mainly by inhalation in a concentration-dependent manner, but is likewise absorbed well via oral and dermal routes. The main excretory pathway is the urine. After naphthalene is absorbed by the organism, the substance is metabolized by enzymes to different reactive metabolites; the toxicity depends on the metabolites formed. There are qualitative and quantitative differences in metabolism depending on the species, particularly as regards CYP-catalysed oxidation. The CYP2F enzymes in the respiratory tract of rodents, the target tissue of the toxicity induced by naphthalene, display particularly high enzymatic activity towards naphthalene.

## 4 Effects in Humans

See also Greim (1998). There are no new data for the end points single exposures, local effects on skin and mucous membranes, allergenic effects and reproductive and developmental toxicity.

### Repeated exposure

A cross-sectional study of 61 male employees working in abrasives manufacturing companies (3 in Germany, 2 in Austria) who had not smoked for at least the 12 preceding months investigated the induction of acute sensory irritation or (sub)chronic inflammatory effects in the eyes, nose and respiratory tract after exposure to naphthalene. In the abrasives industry, naphthalene is handled openly and represents the main source of exposure in terms of potential irritation on the respiratory tract. The determinations were carried out during a single working week, pre-shift on Monday and post-shift on Thursday. The nose was examined endoscopically to identify (sub)clinical signs of irritation, inflammation and damage to the nasal mucosa. A questionnaire was completed to detect possible habituation to the odour of naphthalene and to subjectively evaluate the effects of odour and irritation. Sensitivity to smell was assessed by the Sniffin’ Sticks test. Changes to the humoral and cellular composition of blood, nasal lavage fluid (NALF) and induced sputum (IS) were likewise investigated. On the basis of the data obtained by personal air monitoring and biological monitoring (sum of 1-naphthol and 2-naphthol in the urine) as well as the workers’ occupational history, the participants were divided into a group of highly exposed persons (n = 22, direct exposure during the activities mixing, sieving, moulding and pressing), a group of moderately exposed persons (n = 17, bystander exposure, occasional exposure in areas in close proximity to the workstations) and a reference group (n = 22). During the work shift, average naphthalene concentrations of 0.15 mg/m^3^ (median: 0.13 mg/m^3^; range: 0.05–0.36 mg/m^3^) were determined in the reference group, 0.66 mg/m^3^ (median: 0.59; range: 0.20–1.22) in the group with moderate exposure and 6.97 mg/m^3^ (median: 6.30; range: 2.46–11.58) in the group with high exposure. Individual short-term values of up to 145.8 mg/m^3^ were determined in the high exposure group, particularly in the workers engaged in sieving. Determinations taken at an earlier time point (no other details) revealed low concentrations of dust (inhalable fraction ≤ 5.5 mg/m^3^, respirable fraction ≤ 1.0 mg/m^3^) in 3 companies and concentrations of crystalline silica of up to 100 µg/m^3^ in 2 companies. The biological monitoring involved the collection of daily urine samples from Monday to Thursday before and after the shift; reference group samples were taken only on Monday and Thursday. The naphthol concentrations (cumulative value) in the urine samples taken after the shift were in the range of < 1 to 10 127 µg/l and showed a linear correlation with the naphthalene concentration in the air. Additionally, the cumulative naphthol levels increased in a sawtooth pattern over the course of the working week. In some cases, workers with exposure to naphthalene exceeded the reference value (BAR) of 35 µg/l determined in persons of the general population without occupational exposure even after a work-free weekend. The naphthol levels excreted in the urine returned to the range of the BAR value only after a period with no exposure to naphthalene of at least 2 weeks (for example, holidays). The authors concluded that there is no longer lasting naphthalene storage effect in the organism.

The workers were of a wide range of ages. The mean age of the persons in the high exposure group was 39.4 (25–58) years, that of the moderate exposure group was 46 (24–60) years and that of the reference group was 46 (23–62) years. The mean duration of exposure in the high exposure group was 6.8 (0.3–21.8) years and thus somewhat lower than that of the moderate exposure group of 9.1 (0.4–33.9) years and that of the reference group of 9.0 (0.6–34.4) years.

Chronic diseases such as thyroid disorders, hypertension or increased cholesterol levels, disorders of the nose and respiratory tract such as chronic rhinitis, bronchitis or asthma and respiratory allergies, atopy or a partial loss of smell did not occur more frequently among persons with exposure to naphthalene than among the reference group. The high exposure group reported an increase in work-related complaints of the eyes and nose that was statistically significant in comparison with the incidence found in the reference group. The moderate exposure group described complaints in the nose, but none in the eyes. The workers of the high exposure group attributed their eye complaints to exposure to naphthalene, while the workers in the reference group attributed them to screen work. The workers of the high exposure group further reported that the irritation in the eyes and nose was noticeable only when they were handling naphthalene directly. The authors concluded that the eye complaints were subjective complaints that do not fulfil the criterion for adverse sensory irritation.

In general, the workers described the odour of naphthalene as intense and unpleasant. No differences were observed between the exposure groups and no habituation effects developed during the week of the study. The workers of the high exposure group described themselves as less sensitive to smells and chemicals in comparison with the other groups. The endoscopic examination of the nose carried out pre-shift on Monday did not reveal (sub)clinical signs of irritation, inflammation or damage to the nasal mucosa. At the end of the shift on Thursday, slight swelling of the nasal mucosa and a slight increase in secretions were observed in the 2 exposure groups in comparison with the findings in the reference group. However, the 2 exposure groups did not differ with statistical significance from one another and no concentration–effect relationships were observed with respect to an increase in the number or severity of the inflammatory effects with increasing exposure.

The cellular and humoral parameters determined in the serum, NALF and IS, did not differ with statistical significance between the 3 groups. Other than reduced levels of club cell secretory protein (CC16) in the serum on Thursday, the samples collected pre-shift on Monday did not differ from those collected post-shift on Thursday. The authors concluded that a consistent pattern of inflammatory effects induced by exposure to naphthalene was not evident and no concentration dependency was found.

For most of the investigated parameters, no differences were found between the persons in the moderate and high exposure groups and adverse changes were not observed over the course of the week. The authors called into question that the slight differences that were observed can be attributed to exposure to naphthalene in view of the marked differences in exposure levels. Overall, after mean long-term exposure to concentrations up to about 7 mg/m^3^ and in some cases marked short-term excursions, there were no effects that could be clearly associated with exposure to naphthalene (Sucker et al. 2017, 2021; Weiss et al. 2020).

### Reproductive toxicity

The evaluation of the Committee on Hazardous Substances (Ausschuss für Gefahrstoffe, AGS) includes several studies of fertility. The studies found an inverse correlation between sperm motility and quantity and the concentrations of 1-naphthol, 1-hydroxynaphthalene or 1-hydroxynaphthalene and 2-hydroxynaphthalene (AGS 2018).

The documentation from 1995 describes transplacental naphthalene intoxications. In both cases, the mothers had chewed moth balls containing naphthalene over a long period of time during the last trimester of pregnancy. Haemolytic anaemia with jaundice occurred in the newborn babies 7 hours and 3 days after birth, respectively (Greim 1998). There are case studies that found haematological effects in newborn babies after maternal exposure to naphthalene, but no epidemiological data (AGS 2018). Two recent studies reported haematological effects induced by chewing moth balls during the second and third trimester (Sahni et al. 2019; Shafer et al. 2020).

By applying linear models, Nie et al. (2018) established an association of 2-hydroxynaphthalene concentrations in the urine of 263 pregnant women who delivered children with lower birth weights (–4.38% for the high exposure group in comparison with the low exposure group for 2-hydroxynaphthalene; p (trend test) = 0.049) and a higher cephalization index (+4.30% for the high exposure group in comparison with the low exposure group for 2-hydroxynaphthalene; p (trend test) = 0.038).

### Genotoxicity

Chromosomal aberrations and translocations in the blood lymphocytes of 113 children living in urban environments correlated with statistical significance with the concentrations of 1-naphthol and 2-naphthol in the urine (Orjuela et al. 2012). However, the increase in chromosomal aberrations cannot be attributed to exposure to naphthalene because there may have been exposure to additional compounds that induce these changes such as other PAHs (Bailey et al. 2016). The study has therefore not been included in the evaluation.

In 2 other studies, an increased incidence of DNA damage (comet assay) in the sperm correlated with increased levels of 2-naphthol in the men’s urine (Han et al. 2011; Meeker et al. 2007). However, it is assumed that other environmental chemicals contributed to the development of the observed effects. It is therefore not possible to attribute the DNA damage that was observed to exposure to naphthalene.

In a comet assay carried out in US Air Force workers who were exposed to jet propulsion fuel (JP-8), a correlation between DNA strand breaks in the lymphocytes and exposure to naphthalene was not established. The naphthalene concentrations in the air determined by personal air monitoring were 2.7 ± 2.7 µg/m^3^ (range: 0.67–16.88 µg/m^3^) in the low exposure group (n = 138), 63.6 ± 174.6 µg/m^3^ (range: 0.67–932.13 µg/m^3^) in the moderate exposure group (n = 37) and 643.9 ± 682.2 µg/m^3^ (range: 0.67–3910.82 µg/m^3^) in the high exposure group (n = 115) (Krieg et al. 2012).

Naphthalene is a constituent of bitumen. In studies of (oxidative) DNA damage carried out in persons with exposure to bitumen, no relationship was found between naphthalene metabolites in the urine and the effects observed in humans (Nies et al. 2022).

### Carcinogenicity

Carcinogenic effects could not be derived for naphthalene from the data currently available for humans (Greim 1998; Hartwig 2012; IARC 2002). This was confirmed by a re-evaluation of the epidemiological data (Bailey et al. 2016; Lewis 2012).

An evaluation of several studies that investigated the lung cancer risk for workers in occupations with probable exposure to naphthalene was not able to establish a relationship between exposure to naphthalene and cancer of the lungs or nose. Exposure to petroleum, asphalt, creosote and jet fuel were taken into consideration. The author noted that all studies were of limited validity because of shortcomings such as a lack of exposure data, low levels of naph­thalene exposure, exposure to other chemical substances and a small number of cases. Therefore, the weak data do not make it possible to rule out a possible association (Lewis 2012).

The nasal tumours of the olfactory neural epithelium observed in rats after inhalation exposure are a form of tumour that occurs very rarely in humans. Fewer than 1000 cases worldwide were reported up to the year 2000 (AGS 2018). In the United States, 910 nasal tumours were observed per year over the time period from 1973 to 2006, including 66 olfactory neuroblastomas. In comparison, 65 905 rare nasal tumours, including 29 121 olfactory epithelial neuroblastomas, were predicted annually for the US based on the unit risk factors derived for naphthalene by the US EPA from an NTP study with rats. The authors therefore recommend that the risk assessment published by the US EPA should be re-evaluated (Magee et al. 2010).

## 5 Animal Experiments and in vitro Studies

### 5.1 Acute toxicity

The following studies have been published since the last addendum from 2001 (Hartwig 2012).

#### 5.1.1 Inhalation

After groups of 3 male Sprague Dawley rats were exposed to naphthalene concentrations of 0, 3.4 ± 0.5 or 23.8 ± 1.7 ml/m^3^ for 4 hours, naphthalene induced damage to the olfactory epithelium in a concentration-dependent manner after 24 hours (Lee et al. 2005).

F344 rats and Sprague Dawley rats (5 rats per sex and strain) were exposed to naphthalene concentrations of 0, 0.1, 0.3, 1, 10 or 30 ml/m^3^ for 6 hours. Necrotic changes in the olfactory epithelium were noticeable at concentrations of 0.1 ml/m^3^ and above in Sprague Dawley rats and at 1 ml/m^3^ and above in F344 rats. Their incidence increased with the concentration. This damage was evident in all exposed rats at concentrations of 10 ml/m^3^ and above. In the respiratory epithelium of the nose, necrosis occurred at higher concentrations than in the olfactory epithelium and was characterized by cytoplasmic vacuolation, pyknotic nuclei and sloughing of the necrotic epithelium. Little inflammatory cell infiltration was present. No sex-specific differences were noticeable, but Sprague Dawley rats showed greater sensitivity than F344 rats (Dodd et al. 2010).

### 5.2 Subacute, subchronic and chronic toxicity

The following studies have been published since the last addendum from 2001 (Hartwig 2012).

#### 5.2.1 Inhalation

F344 rats and Sprague Dawley rats (5 to 10 rats per sex and strain) were exposed to naphthalene concentrations of 0, 0.1, 1 or 10 ml/m^3^ for 6 hours a day, on 5 days. Additionally, 10 rats of the high concentration group were observed for 14 days after the end of exposure. Degenerative necrotic changes in the olfactory nasal epithelium were noticeable in female Sprague Dawley rats at concentrations of 0.1 ml/m^3^ and above and in male Sprague Dawley rats and in F344 rats of both sexes at concentrations of 1 ml/m^3^ and above. Their incidence increased with the concentration. The lesions included cytoplasmic vacuolation, condensation of the cytoplasm, pyknotic and karyorrhectic nuclei, loss of proper epithelial orientation and the thinning and sloughing of the epithelium. Areas of re-epithelialization were noted beneath the sloughed epithelium but were not classified as metaplasia. Only minimal inflammatory cell infiltration was observed. Additionally, basal cell hypertrophy or hyperplasia of the nasopharyngeal goblet cells was observed in animals of the high concentration group. After recovery for 14 days, the lesions induced by the naphthalene concentration of 10 ml/m^3^ were weaker, but still detectable (Dodd et al. 2010).

Ten female and 10 male F344 rats per concentration group were exposed by inhalation to naphthalene concentrations of 0, 0.1, 1, 10 or 30 ml/m^3^ for 6 hours a day, on 5 days per week, for 90 days, with subsequent histopathological examination of the nose. Ten additional animals per sex and concentration were examined 4 weeks after the end of exposure. Six cross-sections were taken of the nasal cavity. No changes were induced by naphthalene in the rats of the low concentration group. Minimal hyperplasia was detected in the transitional/respiratory epithelium at 1 ml/m^3^ (section level 2; grade of severity 1 on a scale of up to 5); this effect was observed also in the higher concentration groups (section level 2; grade of severity 1.4). Additionally, basal cell hyperplasia and degeneration/necrosis in the olfactory epithelium and goblet cell hyperplasia in the nasopharyngeal tract were noticeable at 10 and 30 ml/m^3^ (levels 2–5; grade of severity 1–2.7). Four weeks after the end of exposure, all effects were either less severe or completely reversible with the exception of the basal cell hyperplasia and degeneration/necrosis in the olfactory epithelium found in the 2 high concentration groups (Dodd et al. 2012; see Table 1).

Additionally, gene expression analyses were carried out in the nasal tissue after 90 days; these found signs of oxidative stress, inflammation and proliferation (see Section 2.2; Clewell et al. 2014).

As shown in Table 1, inhalation exposure of male F344 rats to naphthalene caused lesions in the nose; these effects were dependent on the concentration and time. The results for female rats were similar (data not shown) (Dodd et al. 2010, 2012; NTP 2000).

**Tab.1:** Comparison of non-neoplastic changes in the nasal cavity of male F344 rats exposed to naphthalene (according to AGS 2018; Dodd et al. 2010, 2012; NTP 2000)

Cell type / lesion	Naphthalene [ml/m^3^]									
	0.1		1		10			30		60
	5 days	90 days	5 days	90 days	5 days	90 days	2 years	90 days	2 years	2 years
RE hyperplasia	N	N	N	10/10 (1.0)^a)^	N	10/10 (1.4)	21/49 (2.2)	10/10 (1.4)	29/49 (2.0)	29/49 (2.2)
RE squamous metaplasia	N	N	N	N	N	8/10 (0.9)	15/49 (2.1)	8/10 (0.8)	23/48 (2.0)	18/48 (1.8)
OE degeneration	N	N	8/10 (0.8)	N	10/10 (2.8)	10/10 (1.6)	46/49 (1.7)	10/10 (2.2)	40/48 (1.7)	38/48 (1.5)
OE hyperplasia	N	N	N	N	N	9/10 (0.9)	48/49 (2.1)	10/10 (1.9)	45/48 (2.5)	46/48 (3.0)

a) Number with effects/number of examined animals (mean level of severity: 1 = minimal, 2 = mild, 3 = moderate, 4 = severe); N: no lesions induced by treatment; OE: olfactory epithelium; RE: respiratory epithelium

#### 5.2.2 Oral administration

Naphthalene given in oral doses of 0, 49, 98 or 147 mg/kg body weight and day to groups of 3 male Wistar rats for 14 days led to an increase in the mean total protein concentrations in the serum. The activity levels of alanine aminotransferase (ALT), aspartate aminotransferase (AST) and alkaline phosphatase (ALP) were not increased with statistical significance (Chukwunonyelum et al. 2016).

### 5.3 Local effects on skin and mucous membranes

Naphthalene causes weak irritation of the skin and eyes of rabbits (Greim 1998).

### 5.4 Allergenic effects

A modified maximization test in guinea pigs did not find evidence of sensitizing effects induced by naphthalene. Intradermal and topical induction were carried out either with a 1% formulation in paraffinum liquidum (white mineral oil) or with a 10% formulation in petrolatum. Topical induction was preceded by treatment with 10% sodium lauryl sulfate in petrolatum for 24 hours. The challenge treatment after 24 hours was carried out with 0.1% and 1% formulations in acetone. None of the 12 female Hartley guinea pigs produced a response at the readings taken after 24 and 48 hours (Okada et al. 1985).

A Buehler test with undiluted naphthalene likewise did not produce a reaction in 20 guinea pigs (EU 2003).

### 5.5 Reproductive and developmental toxicity

The studies of developmental toxicity in rats, mice and rabbits included in the documentation from 1995 did not provide evidence of teratogenicity (Greim 1998).

The publicly accessible ECHA registration dossier includes a large number of studies of fertility and developmental toxicity that were either unpublished or not designated as published studies. In all of these studies, negative or not significant results were obtained in histological analyses for the effects on the reproductive organs (male and female animals, rats, mice and rabbits). Relevant evidence was not reported by studies of fertility and teratogenicity carried out under the NTP programme (AGS 2018).

### 5.6 Genotoxicity

As one objective of this addendum is the re-evaluation of genotoxicity, all available studies are presented in detail in the section below.

#### 5.6.1 In vitro

An overview of the data from in vitro studies of genotoxicity is shown in Table 2.

In numerous mutagenicity tests in the Salmonella typhimurium strains TA97, TA98, TA100, TA1535, TA1537, TA1538, UTH8413 and UTH8414, genotoxic and mutagenic effects were not induced by naphthalene up to cytotoxic concentrations either with or without metabolic activation (Bos et al. 1988; Connor et al. 1985; Florin et al. 1980; Gatehouse 1980; Ho et al. 1981; Kaden et al. 1979; McCann et al. 1975; Mortelmans et al. 1986; Narbonne et al. 1987; NTP 1992; Sakai et al. 1985; Schreiner 2003; Seixas et al. 1982). However, these strains are used to detect changes only to the bases guanine and cytosine and not mutagenic processes involving changes to adenine or thymine. These processes would require testing in the Salmonella typhimurium strains TA102, TA104 or Escherichia coli WP2 that are sensitive also for oxidative damage.

Naphthalene yielded negative results in the test for DNA repair and prophage induction and in the 8-azaguanine resistance test in Salmonella typhimurium and Escherichia coli (Ho and Ho 1981; Mamber et al. 1983, 1984; Nakamura et al. 1987; Schreiner 2003). A positive mutagenicity test in Salmonella typhimurium TA1535 (Narbonne et al. 1987) is not regarded as of importance in light of the large number of mutagenicity tests in Salmonella typhimurium, including TA1535, that yielded negative results.

Naphthalene induced a concentration-dependent increase in sister chromatid exchange (SCE) in Chinese hamster ovary (CHO) cells both with and without the addition of metabolic activation from rat liver. The data available for cytotoxicity are imprecise; the highest concentration was limited by cytotoxicity. However, SCE was induced even below the highest concentration (NTP 1992).

SCE was not detected in human lymphocytes after the administration of 100 µM naphthalene for 2 hours both with and without the addition of metabolic activation by human liver microsomes. Naphthalene did not induce statistically significant levels of cytotoxicity in these studies (Tingle et al. 1993; Wilson et al. 1995).

In mouse macrophages (J774A.1), reactive oxygen species, DNA fragmentation and lipid peroxidation were increased concurrently with cytotoxicity at naphthalene concentrations of 200 µM and above (Bagchi et al. 1998 b).

Treatment of rat hepatocytes with 0 to 3 µM of naphthalene did not increase the incidence of DNA strand breaks up to cytotoxic concentrations in the alkaline elution assay. Cytotoxicity was evaluated by glutamate oxaloacetate transaminase release or by trypan blue dye exclusion (Sina et al. 1983). The first test is not regarded as valid because of marked variations.

In the TUNEL test, naphthalene induced a statistically significant increase in DNA fragments in human lymphocytes at all tested concentrations of 10, 25, 50 and 100 µM. Cytotoxic effects were determined using a lactate dehydrogenase test and a cell proliferation test (Kapuci et al. 2014). The results varied extensively, overlapping both the positive and negative control values. The number of samples was not given for any of the tests. As a result, the TUNEL test has not been included in the evaluation.

DNA binding was observed after in vitro treatment of lung explant cultures from 6 mice, 6 rats and 2 rhesus monkeys (animal strains and sex not specified) and nasal explant cultures from rats with 2.5, 25 or 250 µM ^14^C-naphthalene (according to a computational model (Morris 2013), the concentration of 250 µM corresponds to inhalation exposure to a naphthalene concentration of 10 ml/m^3^). The binding level was dependent on the concentration. The highest DNA binding activity (all values estimated from the graphs) was detected in the lungs of mice, the second highest in rhesus monkeys. In all tissues from rats, the DNA binding was lower by a factor of about 60 to 300 than the binding levels determined in the lungs of mice. No correlation was established between DNA binding and tumour incidence in rats because the lung tissue (non-target organ) exhibited about the same or higher DNA binding than the respiratory epithelium of the nose (target organ in male rats) and the olfactory epithelium of the nose (target organ in female rats). At the same concentration, binding of naphthalene to DNA occurred at a similar or higher level (up to ~10 times as high) than the binding to the positive control BaP in both rodent species. Data for statistical significance were not provided (Buchholz 2016). Even though the author uses the term “DNA adducts”, undigested DNA was analysed and the type of DNA binding was not determined in these experiments. As the calculations were not described, the statement “adducts/DNA molecule” is not comprehensible. The validity of the finding of DNA binding in the lungs of rhesus monkeys is limited also by the small number of animals (n = 2). One objective of the study that was not achieved was the identification of the DNA adducts by liquid chromatography/tandem mass spectrometry or accelerator mass spectrometry (LC-MS-AMS or LC-AMS). According to the author, the method requires further development. Additionally, the report does not clearly describe which samples were analysed (Buchholz 2016). Overall, it is not possible to evaluate the study in detail because the description of the method has considerable shortcomings.

The same research group later published a study that investigated the binding of naphthalene to the DNA in lung explant cultures of female and male B6C3F1 mice and rhesus monkeys and male Sprague Dawley rats as well as in the respiratory and olfactory epithelium of male Sprague Dawley rats treated ex vivo with ^14^C-naphthalene for 1 hour. Incubation with 0, 25 or 250 µM naphthalene led to a concentration-dependent increase in DNA binding in all rhesus monkey lung explants. Sex-specific differences were observed in rhesus monkeys and mice. A statistically significant increase in the binding of naphthalene to DNA was observed in female mice (4.7 times as high) and rhesus monkeys (2.1 times as high) in comparison with the binding level found in their male conspecifics. When male and female animals were considered together, the highest levels of DNA binding were found in the lungs of rhesus monkeys, followed by mice. In male rats, DNA binding was much lower both in the lungs and in the respiratory and olfactory epithelium. Additionally, the level of DNA binding in rats was close to or below the limit of quantification and the values did not differ with statistical significance (Carratt et al. 2019 a).

In another study, radioactivity was detected also after enzymatic digestion of rhesus monkey DNA to deoxynucleosides, which is a sign of stable DNA binding. In this study, the number of DNA adducts formed by 1,2-naphthoquinone in male and female rhesus monkeys was much larger than that induced by naphthalene (Carratt et al. 2019 a).

The studies of DNA binding carried out by Buchholz (2016) and Carratt et al. (2019 a) observed similar levels of DNA binding in the lungs (non-target organ) and the nasal epithelium (target organ) of rats. Carratt et al. (2019 a) found that DNA binding activity was close to the limit of quantification both in the lungs and in the nasal tissue. The level of DNA binding activity in descending order by species was given by Buchholz (2016) as follows: mice >> rhesus monkeys >> rats, while the order according to Carratt et al. (2019 a) was: rhesus monkeys > B6C3F1 mice >> SD rats. The reasons for this inconsistency cannot be determined because Buchholz (2016) does not specify which rodent strains were used.

To investigate whether repeated in vivo exposure of mice to naphthalene leads to tolerance to the induction of naphthalene–DNA binding in vitro, female NIH Swiss mice were given daily naphthalene doses of 200 mg/kg body weight (in 200 µl corn oil) by intraperitoneal injection for 7 days. The controls were administered only corn oil. After dissection, the airways were treated ex vivo with 250 µM ^14^C-naphthalene and examined for DNA binding. A somewhat lower tendency for DNA binding activity was found after pretreatment in vivo with naphthalene; however, the differences were not statistically significant and intragroup variations were large. Cytotoxicity was not investigated (Buchholz et al. 2019). However, this study likewise used undigested DNA and did not characterise specific adducts.

Cytotoxicity was not investigated by Buchholz (2016) and Buchholz et al. (2019). In the ethidium heterodimer-1 assay, cytotoxic effects in the form of permeable cells were induced in several samples after incubation with naphthalene (Carratt et al. 2019 a). No data were provided for the concentration and type of tissue used or for the number of samples and statistical significance. Reference is made to a similar study that analysed respiratory explants collected by the same method (West et al. 2003). In this study, cytotoxicity was induced by naphthalene in the club cells of male Swiss Webster mice after incubation with 300 µM naphthalene for 2 hours. By contrast, the incubation procedure used by the DNA adduct studies involved the incubation of explant cultures of a different mouse strain with up to 250 µM naphthalene for 1 hour. Therefore, conclusions about cytotoxicity cannot be drawn directly from the study of West et al. (2003) and it cannot be evaluated with certainty whether the formation of DNA adducts is secondary to the cytotoxic effects.

A DNA repair synthesis (UDS) test in the cultured hepatocytes of a male F344 rat treated with naphthalene concentrations of up to 5000 µg/ml yielded negative results. Concentrations of 50 µg/ml and above were cytotoxic (Schreiner 2003).

In a study that is available only as an abstract, after incubation with 0.16 mM naphthalene for 24 hours, the incidence of chromosomal aberrations in a mouse embryo culture system (72 hours after conception) was 10 times as high as the levels found in the controls (30 times with metabolic activation); the incidence was markedly decreased after 48 hours. There are no data for cytotoxicity (Gollahon et al. 1990).

Naphthalene induced chromosomal aberrations in CHO cells only in the presence of metabolic activation (NTP 1992). Specific (chromosomal breaks) and unspecific (pulverized cells) lesions were assessed in sum. For this reason, the test has not been included in the evaluation.

A study used different models to define a NOEC (no observed effect concentration) and a point of departure (PoD) for the induction of micronuclei by naphthalene. Human TK6 lymphoblasts were exposed to naphthalene in a concentration range of 0.0625 to 30 µM with the addition of metabolic activation. As determined by flow cytometry, treatment with naphthalene led to a concentration-dependent, non-linear increase in cytotoxicity and micronuclei. The NOEC and PoD calculated from these values were in the range of 2.5 to 10 µM depending on which method of calculation was used. A NOEC of 10 µM was derived if the criterion for exclusion was at least a doubling of the micronuclei in comparison with the levels found in the solvent controls. A NOEC of 2.5 µM was established by applying the statistical method of one-factor ANOVA with Bonferroni correction and a BMCL_10_ of 3.34 µM by using the benchmark approach (10% change). However, the values in the low concentration range (0.0625 and 0.125 µM) were obtained by carrying out only a single determination in triplicate cultures. Cell viability of at least 80% was found at naphthalene concentrations ≤ 20 µM and was reduced to about 40% after incubation with 30 µM naphthalene. Along with the marked decrease in cell viability, the rate of micronuclei increased exponentially. The cytotoxic and genotoxic effects induced after incubation with naphthalene were completely eliminated even at higher concentrations of up to 500 µM by adding 5 mM GSH, which is approximately equivalent to the physiological concentration of GSH in the human liver (~5–10 mM) (Klaassen 2007, p. 168). Also the cytotoxic and genotoxic effects induced by the positive control cyclophosphamide, which is likewise detoxified via GSH (Hajdinák et al. 2020), were eliminated by the addition of GSH (Recio et al. 2012). As a result, it is not possible to determine the relevance of these results for physiological conditions in humans.

In metabolically competent (CYP1A1, 1A2, 2A3, 2E1, 3A4 and epoxide hydrolase) human MCL-5 lymphoblasts, naphthalene induced CREST-negative micronuclei, but no CREST-positive micronuclei or mutations at the *TK* or *HPRT *gene locus. The tested concentrations reduced the cell viability by up to 60% in the mutation test (40 µg/ml) and by up to 30% in the micronucleus test (30 µg/ml). The positive control BaP increased the incidence of mutations in the *TK* and *HPRT* genes, but yielded negative results in the micronucleus test (Sasaki et al. 1997).

**Tab.2:** In vitro studies of the genotoxicity of naphthalene

End point (test method)	Test system	Concentration	Effective concentration	Cytotoxicity/ Comments	Results		References
					–m. a.	+m. a.	
prophage induction	E. coli GY5027 envA^–^uvrB^– ^(λ); GY4015 *amp*^*R*^	0–2000 µg/plate	–	no data	n. t.	–	Mamber et al. 1984
	E. coli K12 envA^–^ uvrB^–^ (λc\ts857)^a)^	0–0.5 mg/ml	–	no data	–	–	Ho and Ho 1981
differential killing (rec assay)	E. coli WP2/WP100 uvr^–^ recA^–^	≤ 2000 µg/plate	–		n. t.	–	Mamber et al. 1984
SOS response (chromo test)	E. coli WP2/WP3478 (polA^–^), E. coli WP2/WP67 (uvrA^–^, polA^–^)	no data	–	no data	–	–	Mamber et al. 1983
	E. coli PQ37	0, 0.156–10 µg/test	–	no data	–	–	Schreiner 2003
SOS response (umu test)	S. typhimurium TA1535 (pSK1002)	0–83 µg/ml	–	no data	–	–	Nakamura et al. 1987
gene mutation	S. typhimurium TA98, TA100, TA1535, TA1537	0–100 µg/plate	–	no data	–	–	McCann et al. 1975
	S. typhimurium TA98, TA100, TA1535, TA1537^b)^	0, 0.3–100 µg/plate	–	100 µg/plate	–	–	Mortelmans et al. 1986; NTP 1992
	S. typhimurium TA98	0, 0.1–0.5 mg/plate	–	no data	n. t.	–	Ho et al. 1981
	S. typhimurium TA1537, TA1538	0, 10–200 µg/ml	–	≥ 150 µg/ml	n. t.	–	Gatehouse 1980
	S. typhimurium TA98, TA100, TA1535, TA1537	0, 0.03–30 µmol/plate	–	≥ 3 µmol/plate	–	–	Florin et al. 1980
	S. typhimurium TA98, TA100, UTH8413, UTH8414	0, 50–2000 µg/plate	–	2000 µg/plate	–	–	Connor et al. 1985
	S. typhimurium TA98, TA100	no data	–	no data; taped-plate test for highly volatile substances and conventional plate test	–	–	Bos et al. 1988
	S. typhimurium TA97, TA98, TA100	0, 5–250 µg/plate	–	250 µg/plate	–	–	Sakai et al. 1985
	S. typhimurium TM677 (8-azaguanine-resistant)	0–2 mM (no other data)	–	no data	–	–	Kaden et al. 1979
	S. typhimurium TA98, TA100, TA1535, TA1537, TA1538	0, 3–300 µg/plate	–	300 µg/plate	–	–	Schreiner 2003
	S. typhimurium TA98, TA1535	0, 5–1000 µg/plate	TA1535: 5, 10 µg/plate	no data; no concentration–effect relationship	n. t.	(+)	Narbonne et al. 1987
	S. typhimurium TA1537	0–1.6 mM (200 µg/plate)	–	90% cytotoxicity at 1.6 mM	–	–	Seixas et al. 1982
sister chromatid exchange	CHO	0, 2.7–90 µg/ml	–m. a.: ≥ 27 µg/ml, +m. a.: ≥	no data	+	+	NTP 1992
	human lymphocytes (♂)	0, 100 µM (12.8 µg/ml)	–	– (MI and PI); m. a.: human liver microsomes	–	–	Tingle et al. 1993
	human lymphocytes (♂)	0, 100 µM (12.8 µg/ml)	–	– (MI and PI); m. a.: human liver microsomes	–	–	Wilson et al. 1995
DNA strand breaks	cultured mouse macrophage cells (J774A.1)	0–500 µM	≥ 200 µM	≥ 200 µM, determined by trypan blue; also lipid peroxidation and ROS formation	+	n. t.	Bagchi et al. 1998 b
	rat hepatocytes (alkaline elution)	0, 0.03, 0.3, 3 µM	–	0.03 µM: 3%; 0.3 µM: 22%; 3 µM: 100% determined by trypan blue; the cytotoxicity test via glutamate oxaloacetate transaminase is not regarded as valid	–	n. t.	Sina et al. 1983
	human lymphocytes (TUNEL)	0, 10–250 µM	≥ 10 µM	0%; major shortcomings, see text	+	n. t.	Kapuci et al. 2014
DNA binding	tissue explant cultures from the lungs of mice (6/conc.), rats (6/conc.), rhesus monkeys (2/conc.) and from the olfactory and respiratory epithelium of rats (6/conc.) strains and sex not specified	mice: 2.5, 25, 250 µM (0.32, 3.2, 32 µg/ml); rats: 2.5, 250 µM; rhesus monkeys: 25, 250 µM; 1 hour	≥ 2.5 µM	no data; DNA binding increased with the concentration; at 250 µM: mouse > rhesus monkey >> rat	+	n. t.	Buchholz 2016
	lung explant cultures (B6C3F1 mice (♂/♀); Sprague Dawley rats (♂); rhesus monkeys (♂/♀))	25, 250 µM (3.2, 32 µg/ml); 1 hour	≥ 25 µM	cell permeability ↑ (concentrations not specified) (ethidium homodimer-1 assay); DNA binding increased with the concentration; at 250 µM: rhesus monkey > mouse >> rat binding to deoxynucleosides observed in rhesus monkeys	+ (♀ > ♂)	n. t.	Carratt et al. 2019 a
DNA binding (tolerance)	mice (NIH Swiss, ♀)	± pretreatment in vivo: 0, 200 mg naphthalene/kg body weight and day, 7 days, intraperitoneal in vitro: 250 µM ^14^C naphthalene	see text and comments	no data; same level of binding of ^14^C-labelled naphthalene to DNA ± pretreatment	+	n. t.	Buchholz et al. 2019
UDS	cultured rat hepatocytes	0, 0.16–5000 µg/ml	–	markedly cytotoxic at 50 µg/ml and above	–	n. t.	Schreiner 2003
chromosomal aberrations	cultured mouse embryos (ICR)	0, 0.16 mM (20 µg/ml)	0.16 mM	no data	+	+	Gollahon et al. 1990
	CHO	–m. a.: 0, 15–75 µg/ml +m. a.: 0, 30–67.5 µg/ml	+S9: ≥ 30 µg/ml	≥ 90 µg/ml; pulverized cells also counted	–	+	NTP 1992
micronuclei	cultured human lymphoblasts (MCL-5)	0–30 µg/ml (0–0.23 mM) (no other data)	30 µg/ml	30%	+^c)^	n. t.	Sasaki et al. 1997
	cultured human lymphoblasts (TK6)	0, 0.0625, 0.125, 0.25, 0.5, 1, 2.5, 5, 10, 20, 30 µM (0–3.8 µg/ml)	≥ 5 µM (0.64 µg/ml)	5 µM: 0%; up to 20 µM ≤ 20%; up to 30 µM: 60%	n. t.	+	Recio et al. 2012
gene mutation HPRT	cultured human lymphoblasts (MCL-5)	0–40 µg/ml (no other data)	–	60%	–	n. t.	Sasaki et al. 1997
TK^+/– ^mutation test	cultured human lymphoblasts (MCL-5)	0–40 µg/ml (no other data)	–	60%	–	n. t.	Sasaki et al. 1997

a) naphthalene pre-incubated with S9 mix (without bacteria)

b)  liquid incubation test: naphthalene pre-incubated, S9 mix and bacteria

c)  positive results for CREST^–^, negative results for CREST^+^

(+): equivocal; conc.: concentration; m. a.: metabolic activation; MI: mitotic index; n. t.: not tested; PI: proliferation index; ROS: reactive oxygen species; UDS: DNA repair synthesis

#### 5.6.2 In vivo

Data from in vivo studies of genotoxicity are summarized in Table 3.

Treatment with naphthalene concentrations of 1, 5 or 10 mM increased the incidence of somatic mutations and recombinations in a wing mosaic test (SMART) in Drosophila melanogaster. When mutations were analysed in 2 marker genes (*flr3* and *mwh*), the standard cross (ST) revealed a lower incidence of mutations in comparison with a metabolically more competent cross (HB; Oregon-flare) that constitutively expresses high levels of CYP enzymes. However, fewer flies than the minimum number (55) required for the calculation of statistical significance were used in all tests with the ST and at one concentration (10 mM) with the HB (Delgado-Rodriguez et al. 1995).

Hepatic DNA strand breaks were not detected by the alkaline elution assay after 2 oral naphthalene doses of 359 mg/kg body weight were given to female Sprague Dawley rats 21 and 4 hours before sacrifice. An increase in cytotoxicity was not found based on ALT levels (Kitchin et al. 1992).

At examination 12 to 72 hours after administration of a single oral dose of naphthalene of 1100 mg/kg body weight, lipid peroxidation and DNA strand breaks were increased in the liver and brain of female Sprague Dawley rats and C57BL/6NTac mice. After 24 hours, maximum GSH depletion was reached in the liver and brain at 51% and 17%, respectively, of the control values. The authors attribute the findings to oxidative stress (Vuchetich et al. 1996). Under the same study conditions, the loss of the tumour suppressor gene *Trp53* increased the damage response in C57BL/6 mice (Bagchi et al. 2000).

Oral administration of naphthalene doses of 110 mg/kg body weight and day for 120 days likewise induced DNA fragmentation in the liver and brain of female Sprague Dawley rats (Bagchi et al. 1998 a).

After groups of 2 male C57BL6 mice were given a single naphthalene dose of 200 mg/kg body weight by intraperitoneal injection, an increased incidence of DNA double-strand breaks (DSB), determined as phosphorylated γH2AX, was found in the lung tissue on examination after 12, 24, 48 and 72 hours. The increase in γH2AX foci/cell reached its maximum 48 hours after injection and decreased again after 72 hours. The incidence of DSB was increased with statistical significance 24 and 48 hours after injection compared with control levels. Other parameters observed were an increase in the number of cells in the BALF, impaired lung function and histopathological findings of damage in the lung epithelium. The damage in the lung epithelium reached its maximum level after 12 hours and then decreased; therefore, it preceded the damage to the DNA (Karagiannis et al. 2012). However, γH2AX is not a marker specific for substance-induced DSB, but is caused also by apoptotic degeneration (Cleaver et al. 2011; Luczak and Zhitkovich 2018). At the same time, the study is of limited validity due to the small number of animals tested (n = 2 per group).

DNA adducts formed after local application of naphthalene (500 (64.08 µg) or 1200 nmol (153.8 µg) in acetone) to the skin of at least 8 female Sencar mice per group for 4 hours. The depurinating naphthalene–DNA adducts with adenine and guanine (1,2-dihydroxynaphthalene-4-N3Ade and 1,2-dihydroxynaphthalene-4-N7Gua) were analysed by ultraperformance LC-MS/MS and increased with the dose from 0.1 (500 nmol) to 0.51 µmol/mol DNA-P (1200 nmol); adenine adducts constituted the larger fraction in all cases. The formation of stable DNA adducts was quantified by ^32^P-postlabelling and found to be about 0.2 µmol/mol DNA-P irrespective of the dose. The structure of the DNA adducts detected by ^32^P-postlabelling was not further specified. Nucleoside 3-monophosphates were used as the positive controls and incubated either with naphthalene and CYP1A1 as the metabolizing system (+ NADPH) or with 1,2-naphthoquinone. Both substances formed different adducts that were likewise observed in vivo. Overall, adduct levels were similar after dermal application of 500 nmol of 1-naphthol, 1,2-dihydrodiolnaphthalene, 1,2-dihydroxynaphthalene or 1,2-naphthoquinone; however, not all DNA adducts that were caused by naphthalene in vitro and in vivo were observed. Therefore, the DNA adducts detected after application of naphthalene formed also via metabolites other than the 1,2-oxidized naphthalene metabolites (Saeed et al. 2009). In all in vivo postlabelling samples analysed by Saeed et al. (2009), the largest fraction of radioactivity was found in the spot with by far the largest molar mass. The authors suggested that this is a sign of undigested DNA. Therefore, the actual DNA adduct levels may be even higher. The lipid peroxidation products detected in the studies of naphthalene in vitro and in vivo (Bagchi et al. 1998 a, b, 2000) may likewise lead to the formation of DNA adducts that would be characterized by a high molar mass (Hartwig et al. 2020; Martinez et al. 2003). As no valid carcinogenicity studies with dermal application of naphthalene to mice are available (see Section 5.7), no conclusions can be drawn about the possible induction of carcinogenicity by the detected lesions. Dermal application of the metabolite 1,4-naphthoquinone led to the formation of papillomas in mice (Fowler et al. 2018). The lungs were the target organ of toxicity in mice after inhalation exposure and intraperitoneal injection. However, the possible formation of DNA adducts in the lungs may follow a markedly different course.

The binding of naphthalene metabolites (1,2-naphthoquinone) to the DNA and the formation of unstable purine base adducts in the skin were reported in the study with Sencar mice. This is not regarded as evidence that these types of reactions occur also in the target cells and cause mutations there. The cytotoxicity in the skin of mice was not investigated. Therefore, the results are no evidence that DNA adducts form in the subtoxic range (Bailey et al. 2016; see also Section 2).

Negative results were obtained in a UDS test in hepatocytes taken from groups of 3 male Sprague Dawley rats that were administered a single oral naphthalene dose of 0, 600, 1000 or 1600 mg/kg body weight and examined 2 or 14 hours after treatment. Cytotoxic effects were not observed up to 1600 mg/kg body weight (no other details). The positive controls yielded the expected results and the results obtained with the vehicle controls were in the range of the historical controls (Hartwig 2012).

A micronucleus test in the bone marrow of groups of 5 male ICR Swiss mice that were administered a single oral dose of naphthalene of 0, 50, 250, 500 or 1500 mg/kg body weight in olive oil likewise yielded negative results. The examination was carried out 24 hours after administration of the substance. Cytotoxicity was not analysed (Greim 1998). Therefore, it is not possible to determine whether the substance reached the target tissue.

In a micronucleus test carried out according to former OECD Test Guideline 474 (1981) in CD-1 mice given a single naphthalene dose of 0 or 250 mg/kg body weight in corn oil by intraperitoneal injection, the frequency of micronuclei in polychromatic erythrocytes from the bone marrow was likewise not increased. Groups of 5 male and 5 female animals per exposure duration were examined 30, 48 or 72 hours after administration. The control animals were examined only after 48 hours. The ratio of polychromatic to normochromatic erythrocytes was decreased with statistical significance (p = 0.05) after 72 hours. Decreased body tension and activity levels, abnormal gait and lacrimation were observed in several animals (Schreiner 2003).

To investigate the mechanism of naphthalene-induced carcinogenicity in nasal tissue, female and male F344 rats (5 per group) were exposed by inhalation to naphthalene vapour in concentrations of 0, 0.1, 1.0, 10 or 30 ml/m^3^ for 6 hours a day, on 5 days a week, for 90 days. Point mutations from CGT to CAT at *Tp53* codon 271 were analysed; this codon is equivalent to the human *TP53* hotspot codon 273. No statistically significant effects were observed in female rats. In male rats, the incidence of mutations in the respiratory and olfactory epithelium was reduced with statistical significance at the concentration of 30 ml/m^3^ in comparison with the values found in the controls. In general, the incidence of spontaneous *Tp53* mutations in the respiratory epithelium of male rats decreased with an increase in concentration; the authors attributed this to increasing cytotoxicity. Cytotoxicity was detected as slight hyperplasia of the respiratory and transitional epithelium at concentrations of 1 ml/m^3^ and above and increased with the concentration at 10 and 30 ml/m^3^ (Meng et al. 2011). However, the investigated change in base pairs from CGT to CAT would not be the primary mutation pattern expected, neither via a ROS-induced mechanism nor a mechanism involving DNA adduct formation as in the case of PAHs (Kucab et al. 2019; Sanger Institute 2020). Therefore, a mutagenic potential cannot be ruled out for this substance based on this negative result.

**Tab.3:** In vivo studies of the genotoxicity of naphthalene

End point	Test system	Exposure	Result	Cytotoxicity/Comments	References
somatic mutations and recombinations (SMART)^a)^ mwh, flr	0, 1, 5, 10 mM with the feed (medium), 48 hours	+	ST: ≤ 52 wings counted/con­centration HB: 10 mM: 58 wings counted; vehicle: 5% Tween-80, 5% ethanol	Delgado-Rodriguez et al. 1995
DNA damage (alkaline elution), liver	rat, Sprague Dawley, 7 ♀	0, 2 × 359 mg/kg body weight, gavage 4 or 21 hours	–	cytotoxicity: – determined as ALT; lethal for 1/7 animals; ODC induced	Kitchin et al. 1992
DNA SSB (alkaline elution), liver, brain	rat, Sprague Dawley, 4–6 ♀	0, 1 × 1100 mg/kg body weight, gavage, 12, 24, 48 or 72 hours	+	1100 mg/kg body weight: ~50% LD_50_	Vuchetich et al. 1996
DNA fragmentation (spectrophotometry), liver, brain	rat, Sprague Dawley, 4–6 ♀	0, 15, 30, 45, 60, 75, 90, 105, 120 days, 110 mg/kg body weight and day, gavage	+	110 mg/kg body weight: 0.05 LD_50_	Bagchi et al. 1998 a
DNA fragmentation (spectrophotometry), liver, brain	mouse, *Trp53*(+/+): C57BL/6NTac, *Trp53*(–/–): C57BL/6TSG-p53, 4 ♀	0, 1 × 1100 mg/kg body weight, gavage, 12, 24, 48 or 72 hours	+	concurrently with lipid peroxidation and GSH depletion; *Trp53*(–/–) > *Trp53*(+/+)	Bagchi et al. 2000
DNA DSB (γH2AX), lungs	mouse, C57BL6, 2 ♂	0, 1 × 200 mg/kg body weight, intraperitoneal, 12, 24, 48 or 72 hours	+	histopathological damage to the epithelium from 12 hours onwards and DNA SSB notice­able from 24 hours onwards	Karagiannis et al. 2012
DNA binding (DNA adducts, ^32^P-postlabelling and UPLC-MS/MS), skin	mouse, SENCAR, ≥ 8 ♀	0, 1 × 500 or 1200 nmol (64 or 153.8 µg), dermal, 4 hours	+	vehicle: acetone	Saeed et al. 2009
UDS, liver	rat, Sprague Dawley, 3 ♂	0, 1 × 600, 1000 or 1600 mg/kg body weight, gavage, 2 or 14 hours	–		Greim 1998
micronuclei, bone marrow	mouse, ICR Swiss, ≥ 5 ♂	0, 1 × 50, 250, 500 or 1500 mg/kg body weight, gavage, 24 hours	–	1000 PCE analysed; cytotoxicity not examined; 1500 mg/kg body weight: LD_100_	Greim 1998
micronuclei, erythrocytes, bone marrow	mouse, CD-1, 5 ♀, 5 ♂	0, 1 × 250 mg/kg body weight, intraperitoneal, 30, 48 or 72 hours	–	> 250 mg/kg body weight: lethal; 1000 erythrocytes analysed; PCE/NCE significantly decreased after 72 hours	Schreiner 2003
gene mutation (*Tp53*), nasal epithelium	rat, F344, 5 ♀, 5 ♂	13 weeks, 0, 0.1, 1, 10 or 30 ml naphthalene va­pour/m^3^, 6 hours/day, 5 days/week, inhalation	– (♀, ♂)	♂: 30 ml/m^3^ significantly decreased incidence of mutations	Meng et al. 2011

a)  ST and HB: “standard” cross and cross with “high bioactivation capacity”, see text

ALT: alanine aminotransferase; DMSO: dimethyl sulfoxide; DNA DSB: DNA double-strand breaks; DNA SSB: DNA single-strand breaks; GSH: glutathione; NCE: normochromatic erythrocytes; ODC: ornithine decarboxylase; PCE: polychromatic erythrocytes; UDS: DNA repair synthesis; UPLC-MS/MS: ultra performance liquid chromatography coupled with mass spectrometry

#### 5.6.3 Metabolites

**1,4-Naphthoquinone** was not mutagenic in vitro in the Salmonella typhimurium strains TA97, TA98, TA100, TA1535, TA1537 and TA1538 and in Escherichia coli WP2uvrA pKM101, a strain that is sensitive to oxidative damage. However, the metabolite was mutagenic in a test with the Salmonella typhimurium strains TA100, TA104 and TA2637. 1,4-Naphthoquinone likewise yielded positive results in a test with Escherichia coli IC203. This strain is generated from WP2uvrA pKM101 and is deficient for the synthesis of anti-oxidative enzymes induced by oxidative stress such as catalase peroxidase and GSH reductase (Fowler et al. 2018).

Mutations were induced in yeasts only at high levels of cytotoxicity (~5% viability) (Fowler et al. 2018).

Signs that 1,4-naphthoquinone induces clastogenic effects were found in tests for SCE, DNA strand breaks and oxidative base damage (comet assay), chromosomal aberrations and micronuclei (CREST^–^). The substance induced also CREST^+^ micronuclei. 1,4-Naphthoquinone was not mutagenic at the *HPRT* and *TK* locus (Fowler et al. 2018).

In vivo treatment with 1,4-naphthoquinone did not have an effect on the formation of chromosomal aberrations or micronuclei in mice or hamsters. Mechanistic studies suggest that the clastogenicity in vitro is caused by the production of ROS; this can be compensated in vivo. However, in vitro findings also suggest the inhibition of topoisomerase II (Fowler et al. 2018).

**1,2-Naphthoquinone** was mutagenic in the Salmonella typhimurium strains TA97, TA100, TA102, TA104 and TA2637 (Flowers-Geary et al. 1996; Hakura et al. 1994). Under redox cycling conditions induced by CuCl_2_, strong mutagenic effects on the tumour suppressor gene *TP53* were obtained with 1,2-naphthoquinone in a mutagenicity test in yeast cells, while no mutagenic effects occurred with 1,2-naphthoquinone alone (Yu et al. 2002 a).

1,2-Naphthoquinone induced sister chromatid exchange in human lymphocytes and γH2AX foci in BEAS-2B cells in vitro (Gurbani et al. 2012; Wilson et al. 1996). DNA damage was detected by the comet assay in MCF7 cells at non-cytotoxic concentrations that concurrently led to the production of ROS and depletion of GSH (Lin et al. 2007). DNA adducts were detected in vitro in lung explant cultures from rhesus monkeys (Carratt et al. 2019 a) and in vivo after dermal application (Saeed et al. 2009).

**1-Naphthol** and **2-naphthol** yielded negative results in mutagenicity tests using different Salmonella and Escherichia coli strains. 1-Naphthol produced positive results in a test for differential killing in Salmonella and Bacillus strains, but negative results in a UDS test with rat hepatocytes. The metabolite did not induce mutations in the TK^+/–^ test with L5178Y mouse lymphoma cells. 1-Naphthol likewise did not cause mutagenic effects in the sex-linked recessive lethal (SLRL) test with Drosophila melanogaster or genotoxicity in the bone marrow of mice or rats in the micronucleus test (reviewed in NTP 1992).

A study that investigated cytotoxic and genotoxic effects (TUNEL test) induced by 1-naphthol and 2-naphthol in human lymphocytes (Kapuci et al. 2014) is not regarded as relevant for the evaluation because the description of the procedure is unclear (number of samples), the sensitivity of the test systems is inadequate (marked variations in the results overlapping both with the positive and negative controls) or the study set-up has shortcomings (lack of a positive control, no data for the standard deviation).

#### 5.6.4 Summary

The available studies in vitro found that naphthalene was not mutagenic in bacteria and human lymphoblasts at cytotoxic concentrations. However, there are no studies available in bacteria strains that are sensitive to oxidative damage and no valid in vitro or in vivo mutagenicity tests in the target cells and tissues of carcinogenicity.

The results of different indicator tests in mammalian cells in vitro revealed genotoxic effects (SCE, UDS, DNA strand breaks). However, this genotoxicity is associated with cytotoxicity and, to a certain extent, with oxidative processes (lipid peroxidation). The DNA strand breaks induced in the liver and brain of mice and rats after oral administration likewise occurred concurrently with increased lipid peroxidation and GSH depletion. DNA strand breaks in the form of γH2AX foci were found in the lungs of mice also after exposure by intraperitoneal injection. The histopathological damage in the lung epithelium was observed prior to the development of the foci. Negative results were obtained in a UDS test in the liver of rats.

In vitro, naphthalene induced micronuclei in human lymphoblasts also at non-cytotoxic concentrations. In vivo tests in the bone marrow of mice after oral administration and intraperitoneal injection produced negative results up to cytotoxic concentrations.

In 3 ex vivo studies, DNA binding activity was observed in lung explant cultures from mice and monkeys. Rhesus monkey explants additionally revealed stable covalent binding to deoxynucleosides. In comparison with these findings, DNA binding activity in rats was much less pronounced and occurred at similar levels in the lung and nose explants. Therefore, the activity levels do not correlate with the tumour incidence. After dermal application to mouse skin, DNA adducts including 1,2-dihydroxynaphthalene adducts with adenine and guanine were likewise detected. Even though the level of DNA binding activity in rat explant cultures does not correlate with the formation of tumours, a mutagenic potential for the observed DNA binding activity cannot be ruled out and a threshold for mutagenic effects cannot be derived because of a lack of valid mutagenicity tests in the target tissue of carcinogenicity in rodents and the very limited number of studies that used primates. As there are no valid carcinogenicity studies with dermal application, the evaluation of specific DNA adducts observed in the skin of mice is likewise not possible. Therefore, it cannot be ruled out at present that naphthalene induces primary genotoxicity.

### 5.7 Carcinogenicity

There are no new animal studies that investigated carcinogenicity. All of the studies described in the earlier evalu­ations (Greim 1998; Hartwig 2012) are presented again below.

#### 5.7.1 Short-term studies

Naphthalene did not induce cell transformation in studies in vitro using the cells of humans, mice, rats or hamsters (Greim 1998; Hartwig 2012).

In rats given naphthalene in 7 subcutaneous injections of 500 mg/kg body weight in sesame oil over a period of 3.5 months, the incidence of lymphosarcomas was increased to 15% (4/34) in comparison with that in the control animals (3%, 1/32) (Knake et al. 1956 in Greim 1998).

In groups of 30 female A/J mice that were exposed by inhalation to naphthalene concentrations of 10 or 30 ml/m^3^ for 6 hours a day, on 5 days a week, for 6 months, the number of adenomas in the lungs per adenoma-bearing mouse, but not the total incidence of animals with lung adenomas, was increased with statistical significance relative to that in the control animals (Adkins et al. 1986 in Greim 1998). The study is not included in the evaluation because of the high spontaneous incidence of lung tumours in the tested mouse strain (Laube et al. 2019).

#### 5.7.2 Long-term studies

##### 5.7.2.1 Inhalation

There are 2 long-term inhalation studies that investigated the carcinogenic effects induced by naphthalene after inhalation exposure of mice and rats. These were described in the earlier documentation (Greim 1998; Hartwig 2012).

B6C3F1 mice were exposed by inhalation for 6 hours a day, on 5 days a week, for 104 weeks to naphthalene concentrations of 0, 10 or 30 ml/m^3^ (Greim 1998; NTP 1992). The control and low exposure groups were composed of 75 animals per sex and the 30 ml/m^3^ group of 150 animals per sex (see Table 4). The mean body weights of the exposed mice were somewhat lower than that of the controls throughout the study period. At the end of the study, the mortality of the male control mice was increased with statistical significance in comparison with the mortality among the exposed mice as a result of wound trauma and secondary infections related to fighting. The mortality of the exposed female mice was similar to that of the controls. In the high concentration group, a statistically significant increase in the incidence of bronchioloalveolar adenomas was observed in female mice. In addition, a single carcinoma was observed in the lungs of one female mouse in the high concentration group. The incidences of adenomas and carcinomas in the lungs were likewise increased in exposed male mice, but not with statistical significance. Non-neoplastic changes were observed primarily in the lungs and nose. Various inflammatory parameters in the lungs were increased in a concentration–dependent manner. Chronic inflammation, hyperplasia of the respiratory epithelium and metaplasia of the olfactory epithelium were detected in the nose of practically all exposed animals, but in only one control animal (NTP 1992).

**Tab.4:** Study of the carcinogenicity induced by naphthalene in B6C3F1 mice after inhalation exposure

Author:	NTP 1992			
Substance:	naphthalene (purity > 99%)			
Species:	**mice**, B6C3F1, 75 ♂, 75 ♀ (0, 10 ml/m^3^); 150 ♂, 150 ♀ (30 ml/m^3^)			
Administration route:	inhalation			
Concentration:	0, 10, 30 ml/m^3^			
Duration:	104 weeks, 6 hours/day, 5 days/week			
Toxicity:	10, 30 ml/m^3^: ♂, ♀: slightly reduced body weights			
		**Exposure concentration [ml/m^3^]**		
		**0**	**10**	**30**
surviving animals	♂	26/75 (37%)**	52/75 (75%)	118/150 (89%)
	♀	59/75 (86%)	57/75 (88%)	102/150 (76%)
**tumours**				
**lungs:**				
bronchioloalveolar adenomas	♂	7/70 (10%)	15/69 (22%)	27/135 (20%)^a)^
	♀	5/69 (7%)	2/65 (3%)	28/135 (21%)*
carcinomas	♂	0/70	3/69 (4%)	7/135 (5%)^c)^
	♀	0/69	0/65	1/135 (1%)^d)^
adenomas and carcinomas	♂	7/70 (10%)	17/69 (25%)	31/135 (23%)^e)^
	♀	5/69 (7%)	2/65 (3%)	29/134 (22%)*
**bone marrow, spleen:**				
haemangiosarcomas	♀	0/69	0/69	5/135 (4%)^g)^
**non-neoplastic lesions**				
**lungs:**				
cellular infiltration: lymphocytes	♂	3/70 (4%)	0/69	8/135 (6%)
	♀	11/69 (16%)	21/65 (32%)	46/135 (34%)*
cellular infiltration: histiocytes	♂	1/70 (1%)	12/69 (17%)*	16/135 (12%)*
	♀	1/69 (1%)	5/65 (8%)	4/135 (3%)
inflammation	♂	0/70	21/69 (30%)**	56/135 (41%)**
	♀	3/69 (4%)	13/65 (20%)*	52/135 (39%)*
granulomatous inflammation	♂	0/70	19/69 (28%)**	15/135 (11%)
	♀	0/69	38/65 (58%)*	42/135 (31%)*
hyperplasia of the alveolar epithelium	♂	2/70 (3%)	7/69 (10%)	15/135 (9%)
	♀	3/69 (4%)	6/65 (9%)	12/135 (9%)
glandular inflammation	♂	7/70 (10%)	14/69 (20%)	22/135 (16%)
	♀	1/69 (1%)	3/65 (5%)	15/135 (11%)*
**nose:**				
inflammation	♂	0/70	67/69 (97%)	133/135 (99%)
	♀	1/69 (1%)	65/65 (100%)	134/134 (100%)
metaplasia of the olfactory epithelium	♂	0/70	66/69 (96%)	134/135 (99%)
	♀	0/69	65/65 (100%)	134/134 (100%)
hyperplasia of the respiratory epithelium	♂	0/70	66/69 (96%)	134/135 (99%)
	♀	0/69	64/65 (98%)	134/134 (100%)

* p ≤ 0.01;

** p ≤ 0.001

a)  historical controls: 6%–24%, 14.4 ± 5.5

b)  historical controls: 0%–10%, 5.8 ± 3.2

c)  historical controls: 0%–14%, 6.3 ± 5.5

d)  historical controls: 0%–6%, 2.8 ± 2.7

e)  historical controls: 10%–30%, 19.7 ± 8.1

f)  historical controls: 0%–12%, 8.4 ± 3.5

g)  historical controls: 0%–6%, 2.6 ± 2.2

In another study, 49 male and 49 female F344 rats were exposed by inhalation to naphthalene concentrations of 0, 10, 30 or 60 ml/m^3^ for 6 hours a day, on 5 days a week, for 105 weeks (NTP 2000). In comparison with the values determined in the controls, the mean body weights of the exposed animals were reduced by less than 10% and survival of the animals was similar in all groups. In the males of all exposure groups, the incidence of adenomas in the respiratory epithelium of the nose was increased both with statistical significance in comparison with the findings in the controls and in the trend test; in the females, these tumours were observed only after exposure to 30 and 60 ml/m^3^ and their incidence did not reach statistical significance. The incidence of neuroblastomas in the olfactory epithelium was increased in the exposed male and female animals (positive results in the trend test): this tumour occurred in the males at concentrations of 30 ml/m^3^ and above and in the females in all concentration groups. In comparison with the values determined in the controls, the incidences were increased with statistical significance only in the females of the high concentration group. Atypical hyperplasia, chronic inflammation and hyalinization developed in the olfactory epithelium. Hyperplasia, squamous metaplasia, hyalinization, goblet cell hyperplasia and glandular hyperplasia were observed also in the respiratory epithelium. In the NTP database of tumour incidences in control animals, neuroblastomas in the nose were reported only for a single female Wistar Han rat in a gavage study carried out in the time period from 2007 to 2008 (NTP 2014). In rats, adenomas of the respiratory epithelium were not observed in any of the control animals. Why the rare neuroblastomas developed in this particular case has yet to be explained (NTP 2000).

##### 5.7.2.2 Dermal application

There was an increase in the incidence of lung adenomas in 25 mice after lifetime dermal application of 0.5% naphthalene dissolved in benzene (no other details). Additionally, 5 tumours observed in the lymphatic system may be attributed to the simultaneous application of benzene. Lung adenomas were seen neither in the untreated animals of this experiment, nor in 170 other control animals (Knake 1956 in Greim 1998). An inhibitory effect on BaP-induced skin tumours was found after a 0.25% naphthalene solution and a 0.003% BaP solution were applied to the skin of ICR/Ha Sprague Dawley mice 3 times a week for 78 weeks (Schmeltz et al. 1978 in Greim 1998).

##### 5.7.2.3 Metabolites

In a study that was carried out in 1940 and cited in a review, dermal application of **1,4-naphthoquinone** dissolved in 1% v/v benzene to the shaved skin of mice (number and strain not specified) for 200 days (daily or every 2 days; no exact data) led to the formation of papillomas in 14 (18%) and to skin cancer in 3 (4%) (no other details) of the 77 surviving mice. The formation of the papillomas was preceded by depilation, keratinization, ulceration and necrosis. In the control group, a papilloma was found in 1 (2%) of 46 surviving animals; skin cancer was not observed in any of the animals. Even though atypical proliferation into deeper layers of tissue and the subcutaneous muscle tissue was described for the papillomas of the group treated with 1,4-naphthoquinone, none of these tumours were transplantable. As no other systemic effects were reported, the authors of the review postulated that the observed skin lesions may have been caused by chronic irritation (necrosis, ulceration) (Fowler et al. 2018).

#### 5.7.3 Summary

Naphthalene does not induce cell transformation in vitro.

The tumours induced by naphthalene in rodents are species-specific with females and males exhibiting different sensitivity. The primary types were malignant neuroblastomas of the olfactory epithelium in female rats, adenomas of the respiratory epithelium in male rats and bronchioloalveolar adenomas in female mice as well as a lung carcinoma in one female mouse in the high concentration group. The incidences of adenomas and carcinomas in the lungs were increased also in male mice; however, the increase was not statistically significant. The simultaneous occurrence of severe inflammation in the relevant target tissues of carcinogenicity and the high incidences of hyperplasia and metaplasia in the rat nose even at the lowest concentration suggest that the carcinogenic effects induced by naphthalene in rats and mice are essentially caused by cytotoxicity resulting from the overloading of detoxifying mechanisms. In spite of species-specific differences in activation, a possible relevance to humans cannot be ruled out (see Section 2).

## 6 Manifesto (MAK value/classification)

The critical effects are the carcinogenic effects induced by naphthalene in the nose of rats and the lungs of mice.

**Carcinogenicity. **After inhalation exposure, malignant neuroblastomas of the olfactory epithelium were observed in female rats, adenomas of the respiratory epithelium in male rats and bronchioloalveolar adenomas in female mice in addition to a lung carcinoma in one female mouse of the high concentration group. In male mice, the incidence of adenomas and carcinomas in the lungs was increased, but not with statistical significance. In spite of species-­specific differences, the tumours that form in rodents may still have human relevance. Valid data from epidemiological studies are not available.

Numerous recent studies and reviews suggest that a non-genotoxic mechanism of action is responsible for the carcinogenic effects induced by naphthalene because the tumours in rats and mice are attributed to increased cell proliferation caused by a severe local inflammatory response to the cytotoxic metabolites of naphthalene. This response occurs only at higher concentrations via binding of the metabolites to proteins after detoxifying mechanisms such as GSH conjugation have been exhausted. Numerous genotoxicity tests obtained positive results only concurrently with cytotoxicity. However, isolated genotoxic effects were observed also at non-cytotoxic concentrations. The role played by the DNA binding activity observed in explants of the target tissue of carcinogenicity in the development of carcinogenic effects cannot be evaluated conclusively because of a lack of valid mutagenicity tests in the target tissue. As there are no valid carcinogenicity studies with dermal application, the evaluation of specific DNA adducts observed in the skin of mice is likewise not possible. Therefore, it is not possible at present to completely rule out a primary genotoxic effect induced by naphthalene. Additionally, there are no studies that can be used to establish an effect threshold for animals or humans. As a result, naphthalene remains classified in Carcinogen Category 2.

**Germ cell mutagenicity. **Naphthalene is not mutagenic in bacteria and human lymphoblasts. However, none of the studies were carried out in strains of bacteria that are sensitive to oxidative damage and there are no valid in vitro and in vivo mutagenicity tests in the target cells/tissues of carcinogenicity.

Positive results in indicator tests in mammalian cells (SCE, UDS, DNA strand breaks) were associated with cytotoxicity and in some cases with oxidative processes (lipid peroxidation). DNA strand breaks induced in vivo in the liver and brain as well as in the lungs occurred either concurrently with an increase in lipid peroxidation and GSH depletion or subsequently to damage to the pulmonary epithelium. In rats, a UDS test in the liver yielded negative results. Naphthalene induced micronuclei in human lymphoblasts in vitro, even at non-cytotoxic concentrations, but not in vivo.

Naphthalene bound to DNA in somatic cells. Even though most genotoxic effects occurred only at cytotoxic concentrations, in isolated cases also below (see Section 5.6.4). There are no studies of germ cell mutagenicity and it is not possible to determine whether the germ cells are reached based on the information available. Therefore, naphthalene remains classified in Germ Cell Mutagen Category 3 B.

**Absorption through the skin. **Several in vivo and in vitro studies are available that documented absorption of naphthalene through the skin on the one hand and adverse effects after dermal application on the other. The fluxes derived from dermal penetration studies in vitro and in vivo are of the same order of magnitude. An in vivo study in humans determined a flux of 0.159 μg/cm^2^ per hour for absorption through the skin. On the basis of this value, about 0.32 mg of naphthalene would be absorbed through the skin after exposure of 2000 cm^2^ of skin for 1 hour. However, a genotoxic potential cannot be ruled out at present, even at this low level of absorption. For this reason, naphthalene remains designated with an “H” (for substances which can be absorbed through the skin in toxicologically relevant amounts).

**Sensitization. **No reliable data are available for sensitizing effects in humans and no positive results from animal studies or in vitro studies. For this reason, naphthalene has not been designated with “Sh” or “Sa” (for substances which cause sensitization of the skin or airways).
